# Supplementary material for: Application of Nitrate, Ammonium, or Urea Changes the Concentrations of Ureides, Urea, Amino Acids and Other Metabolites in Xylem Sap and in the Organs of Soybean Plants (Glycine max (L.) Merr.)
Source: Int J Mol Sci. 2021 Apr 27;22(9):4573. doi: 10.3390/ijms22094573 (PMC8123890; doi:10.3390/ijms22094573)
Supplement: Supplementary file 1 [file ijms-22-04573-s001.zip › ijms-1181694-supplementary.pptx]

## Slide 1
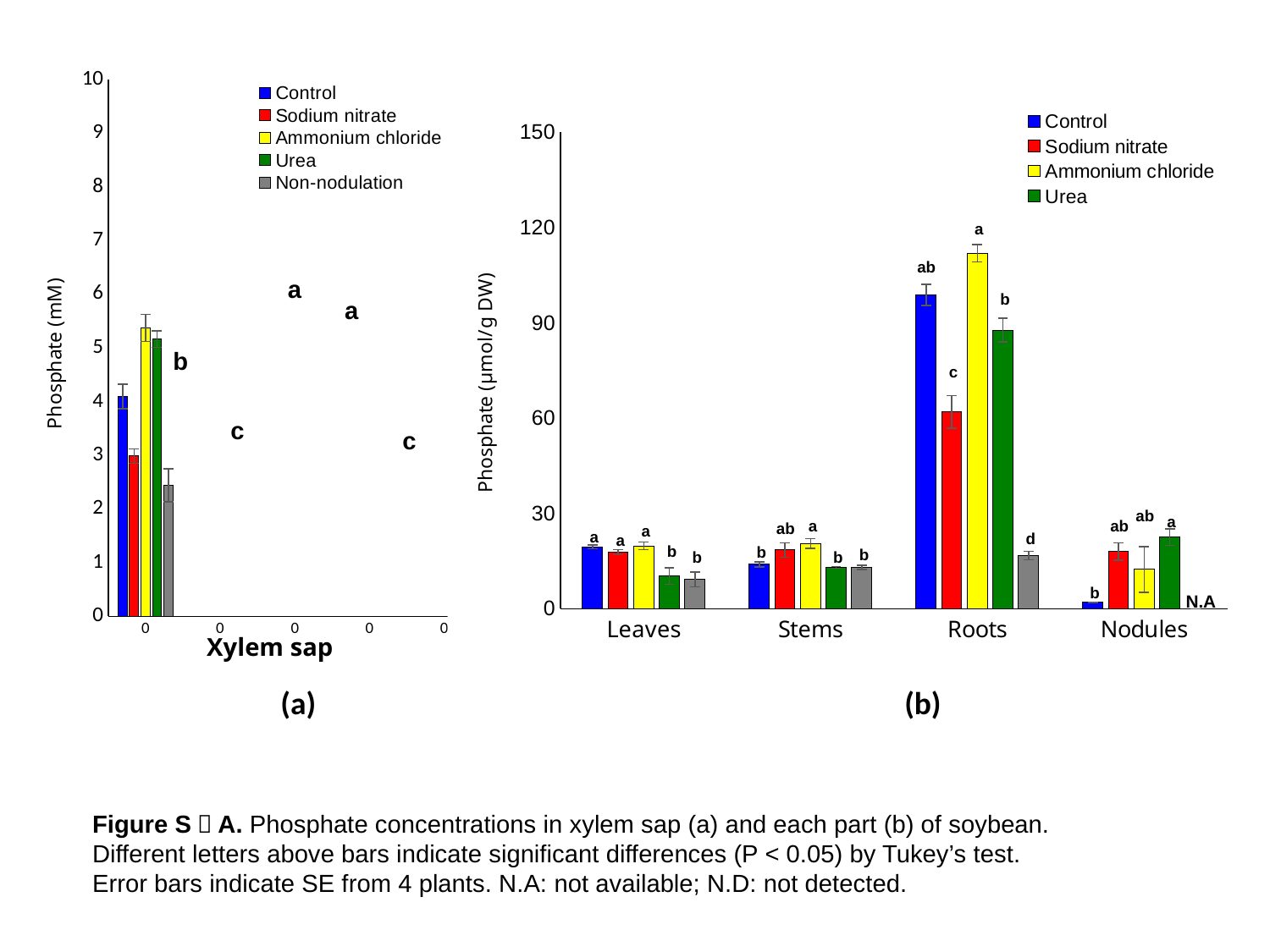

[unsupported chart]
a
a
b
c
c
### Chart
| Category | | | | | |
|---|---|---|---|---|---|
| Leaves | 19.44512401130438 | 17.81270997637442 | 19.79906747152967 | 10.27179356918544 | 9.251949742526273 |
| Stems | 13.99614780241738 | 18.49457665573718 | 20.51038787750526 | 12.99883505571414 | 13.00197719191709 |
| Roots | 98.76262021508072 | 61.94257323787564 | 111.946061982696 | 87.75479782467055 | 16.77014552468384 |
| Nodules | 2.026101220853208 | 18.05282265543522 | 12.4014931775483 | 22.54048678111047 | 0.0 |a
ab
b
c
ab
ab
a
a
ab
a
a
d
a
b
b
b
b
b
N.A
b
Xylem sap
(a) (b)
Figure S１A. Phosphate concentrations in xylem sap (a) and each part (b) of soybean.
Different letters above bars indicate significant differences (P < 0.05) by Tukey’s test.
Error bars indicate SE from 4 plants. N.A: not available; N.D: not detected.

## Slide 2
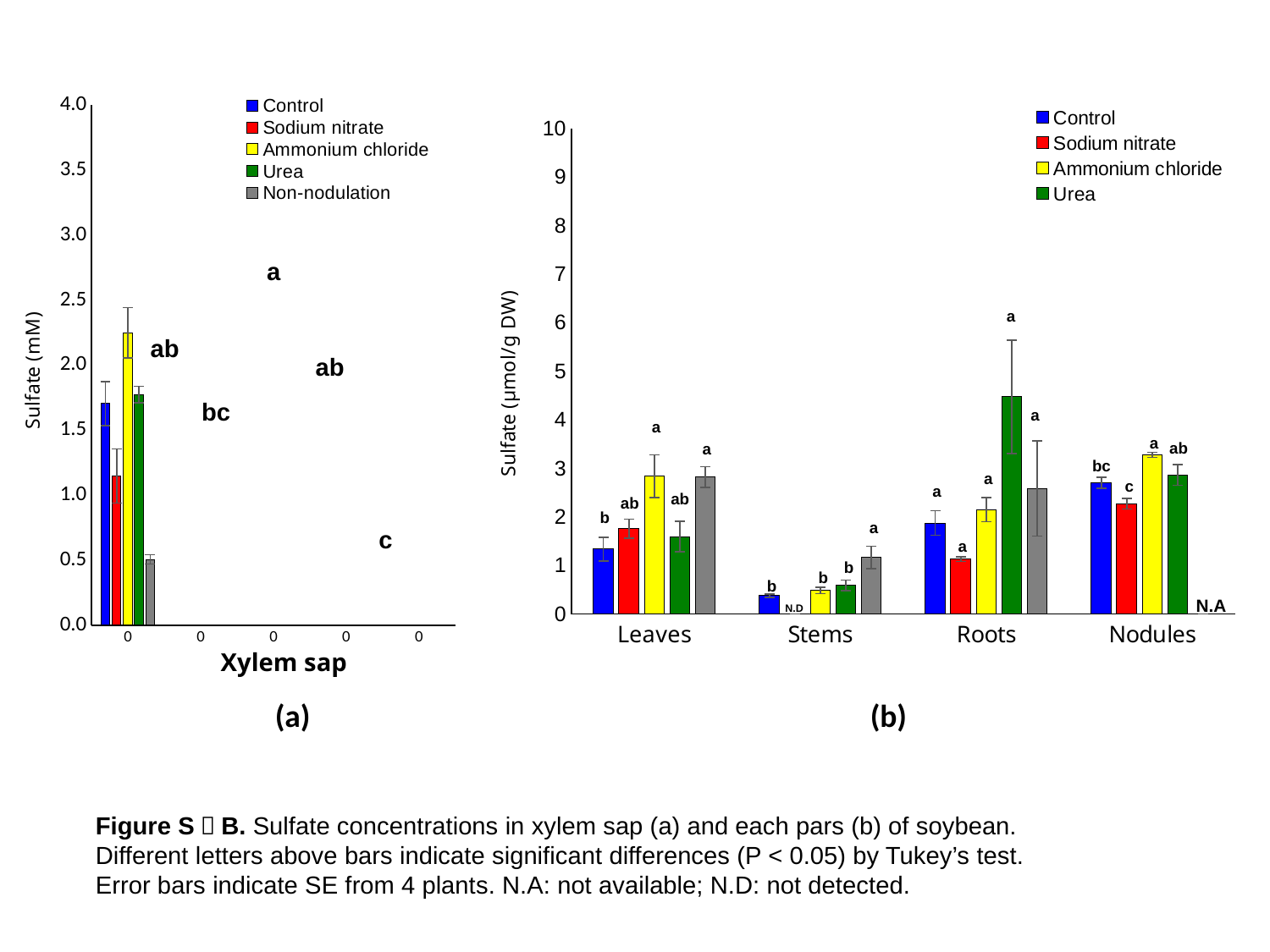

[unsupported chart]
a
ab
ab
bc
c
### Chart
| Category | | | | | |
|---|---|---|---|---|---|
| Leaves | 1.336442211235803 | 1.759658888942547 | 2.838665844271039 | 1.595651424728492 | 2.821617763849895 |
| Stems | 0.378221113636653 | 0.0 | 0.485656438700524 | 0.590909741167502 | 1.164364172251011 |
| Roots | 1.875203531127095 | 1.129062173445744 | 2.149237770666255 | 4.475764264294133 | 2.585188252876027 |
| Nodules | 2.704227239606746 | 2.272457429668596 | 3.279557825938745 | 2.866734265189055 | 0.0 |a
a
a
a
a
ab
bc
a
c
a
ab
ab
b
a
a
b
b
b
N.A
N.D
Xylem sap
(a) (b)
Figure S１B. Sulfate concentrations in xylem sap (a) and each pars (b) of soybean.
Different letters above bars indicate significant differences (P < 0.05) by Tukey’s test.
Error bars indicate SE from 4 plants. N.A: not available; N.D: not detected.

## Slide 3
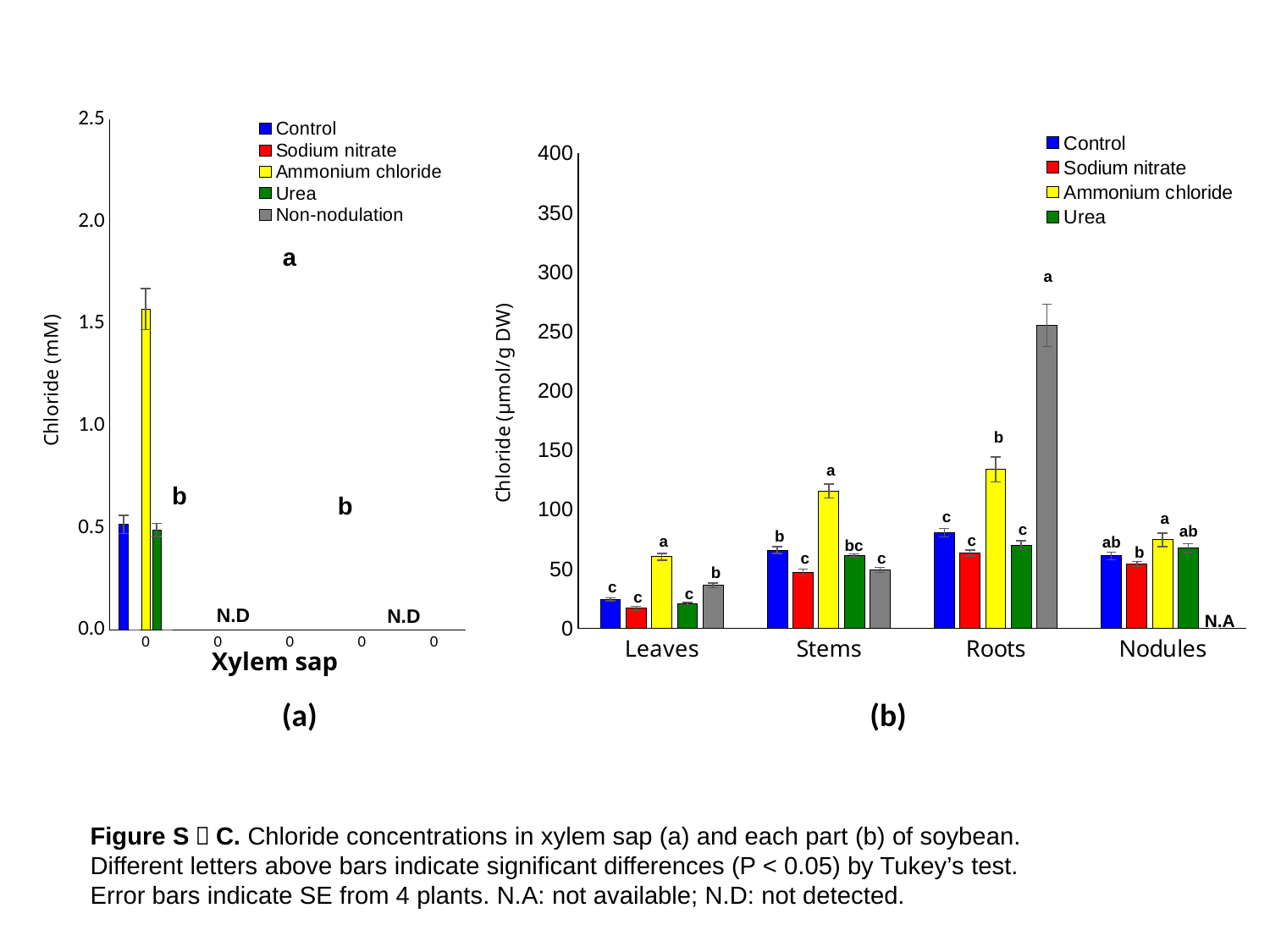

[unsupported chart]
a
b
b
N.D
N.D
### Chart
| Category | | | | | |
|---|---|---|---|---|---|
| Leaves | 24.48880988483088 | 16.90899871537451 | 60.36710947536519 | 20.92234508933752 | 36.17175744129209 |
| Stems | 65.87842690728755 | 47.08159523421596 | 115.7221864803305 | 61.59175867434928 | 49.2358655469752 |
| Roots | 80.5629106188104 | 63.4383897413098 | 133.870510777702 | 70.11846661670258 | 255.3747115349757 |
| Nodules | 61.07851460084323 | 54.28673879584004 | 74.55560611798923 | 67.56260084068245 | 0.0 |a
b
a
c
a
c
ab
b
c
a
ab
bc
b
c
c
b
c
c
c
N.A
Xylem sap
(a) (b)
Figure S１C. Chloride concentrations in xylem sap (a) and each part (b) of soybean.
Different letters above bars indicate significant differences (P < 0.05) by Tukey’s test.
Error bars indicate SE from 4 plants. N.A: not available; N.D: not detected.

## Slide 4
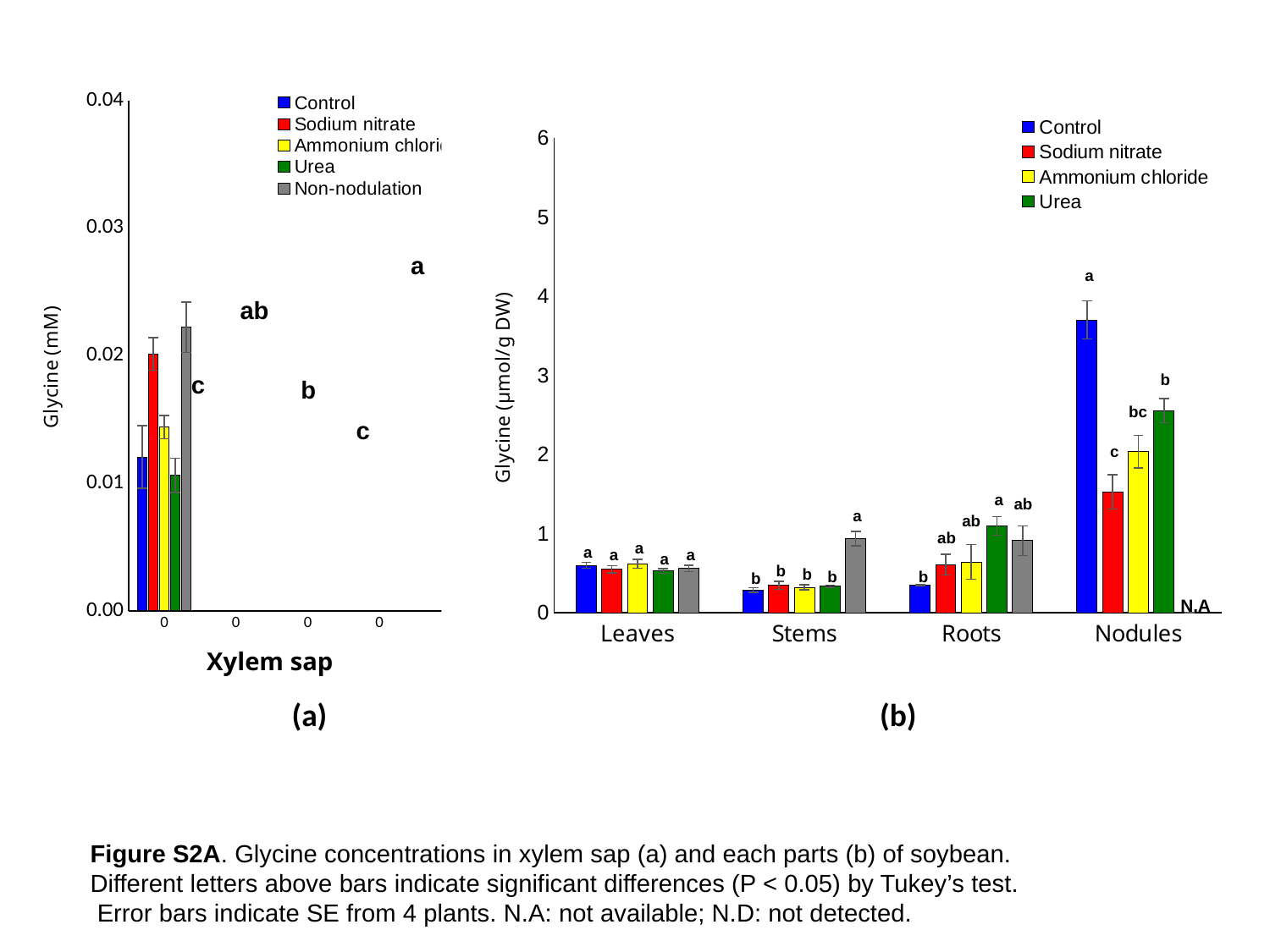

[unsupported chart]
a
ab
c
c
### Chart
| Category | | | | | |
|---|---|---|---|---|---|
| Leaves | 0.599906537336038 | 0.549877547783674 | 0.619344068072746 | 0.530202743959469 | 0.561005464947661 |
| Stems | 0.28863159153174 | 0.347522662338955 | 0.322270479481764 | 0.337617244724534 | 0.938590734559917 |
| Roots | 0.34939951351487 | 0.607623483790817 | 0.642460176012445 | 1.099303241451563 | 0.911221151233535 |
| Nodules | 3.701648404173536 | 1.526761596435033 | 2.034143508552643 | 2.553860088889588 | 0.0 |a
b
bc
c
a
ab
a
ab
ab
a
a
a
a
a
b
b
b
b
b
N.A
Xylem sap
b
(a) (b)
Figure S2A. Glycine concentrations in xylem sap (a) and each parts (b) of soybean.
Different letters above bars indicate significant differences (P < 0.05) by Tukey’s test.
 Error bars indicate SE from 4 plants. N.A: not available; N.D: not detected.

## Slide 5
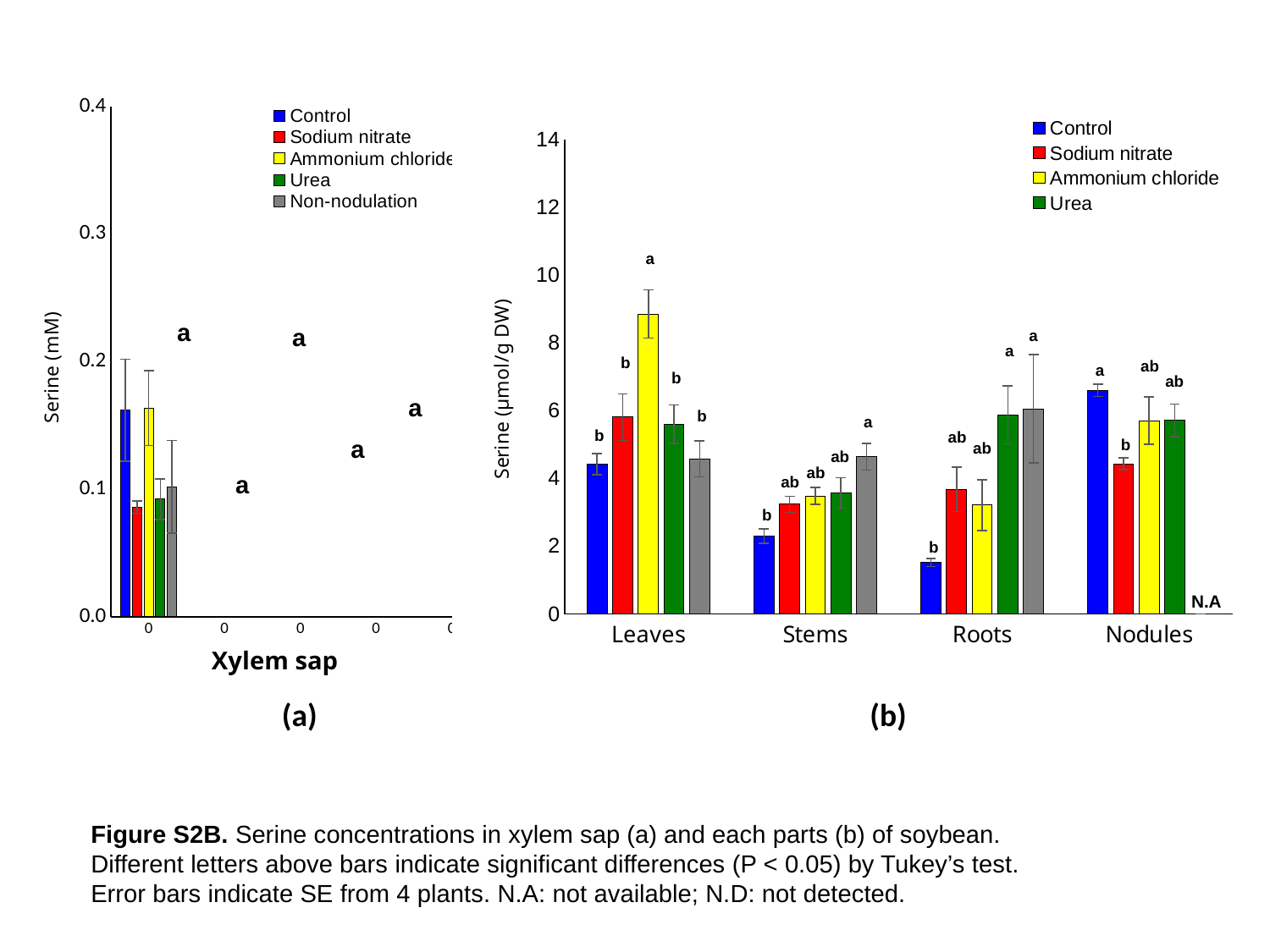

[unsupported chart]
a
a
a
a
a
### Chart
| Category | | | | | |
|---|---|---|---|---|---|
| Leaves | 4.414704979703171 | 5.80268970876417 | 8.84744417408102 | 5.598570883935624 | 4.57451219391986 |
| Stems | 2.29664921804109 | 3.232821119967926 | 3.48057167778973 | 3.564836123408122 | 4.633527780997303 |
| Roots | 1.519114795472219 | 3.676748673081636 | 3.2076071396432 | 5.869594544451115 | 6.05162042347454 |
| Nodules | 6.600470460889367 | 4.424806149189886 | 5.702747307270533 | 5.711805023423132 | 0.0 |a
a
a
b
ab
a
b
ab
b
a
b
ab
b
ab
ab
ab
ab
b
b
N.A
Xylem sap
(a) (b)
Figure S2B. Serine concentrations in xylem sap (a) and each parts (b) of soybean.
Different letters above bars indicate significant differences (P < 0.05) by Tukey’s test.
Error bars indicate SE from 4 plants. N.A: not available; N.D: not detected.

## Slide 6
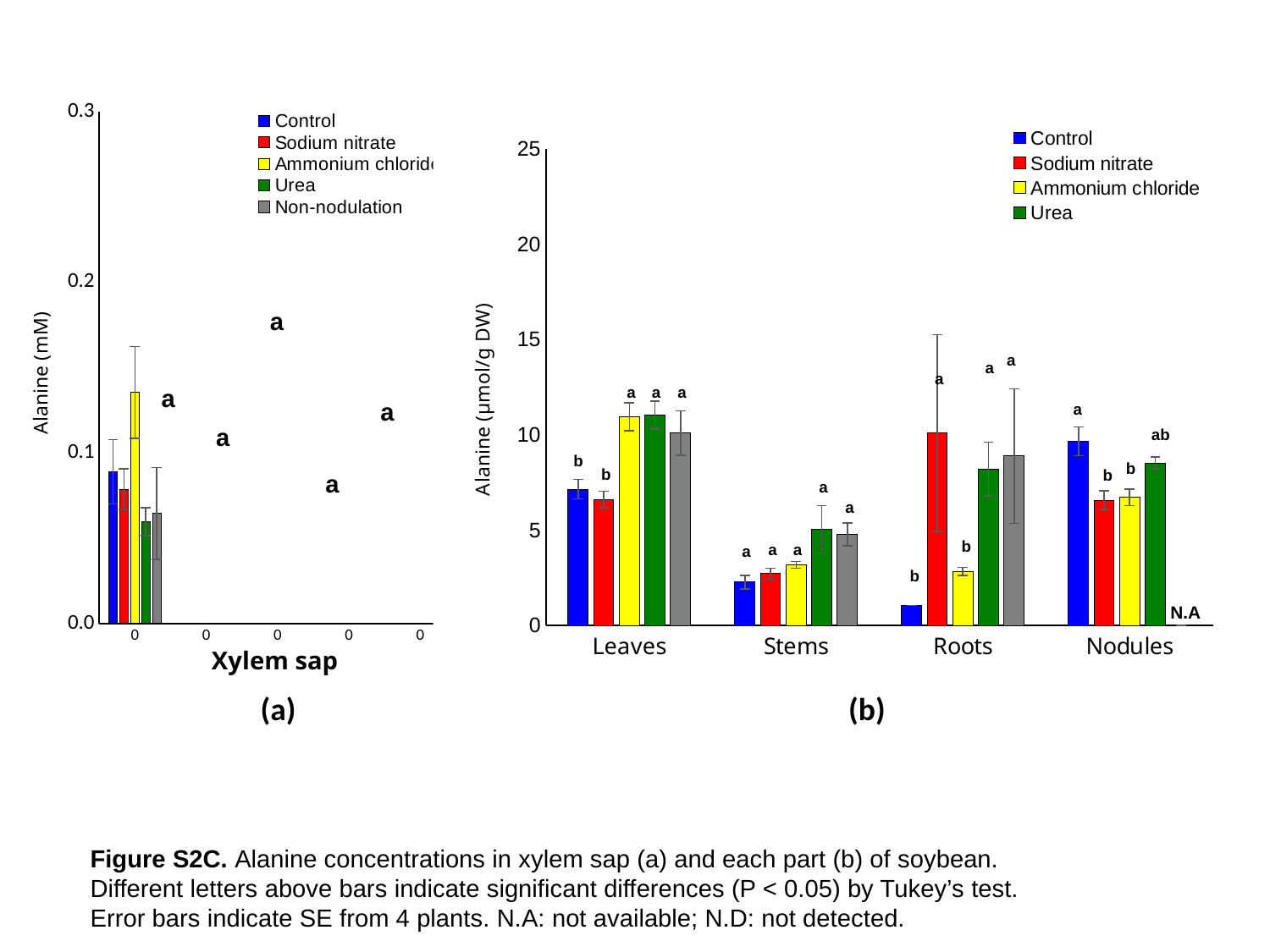

[unsupported chart]
a
a
a
a
a
### Chart
| Category | | | | | |
|---|---|---|---|---|---|
| Leaves | 7.148505817064777 | 6.604710489789784 | 10.9498237747527 | 11.02809211702141 | 10.08881859546302 |
| Stems | 2.267727846953221 | 2.717831786356031 | 3.174503204303927 | 5.03781952134018 | 4.773953314862057 |
| Roots | 1.045068058384865 | 10.1007415233541 | 2.834103383145571 | 8.20944816017655 | 8.882346765079475 |
| Nodules | 9.659191846016382 | 6.567214455731897 | 6.711398009169723 | 8.520681501793849 | 0.0 |a
a
a
a
a
a
a
ab
b
b
b
b
a
a
b
a
a
a
b
N.A
Xylem sap
(a) (b)
Figure S2C. Alanine concentrations in xylem sap (a) and each part (b) of soybean.
Different letters above bars indicate significant differences (P < 0.05) by Tukey’s test.
Error bars indicate SE from 4 plants. N.A: not available; N.D: not detected.

## Slide 7
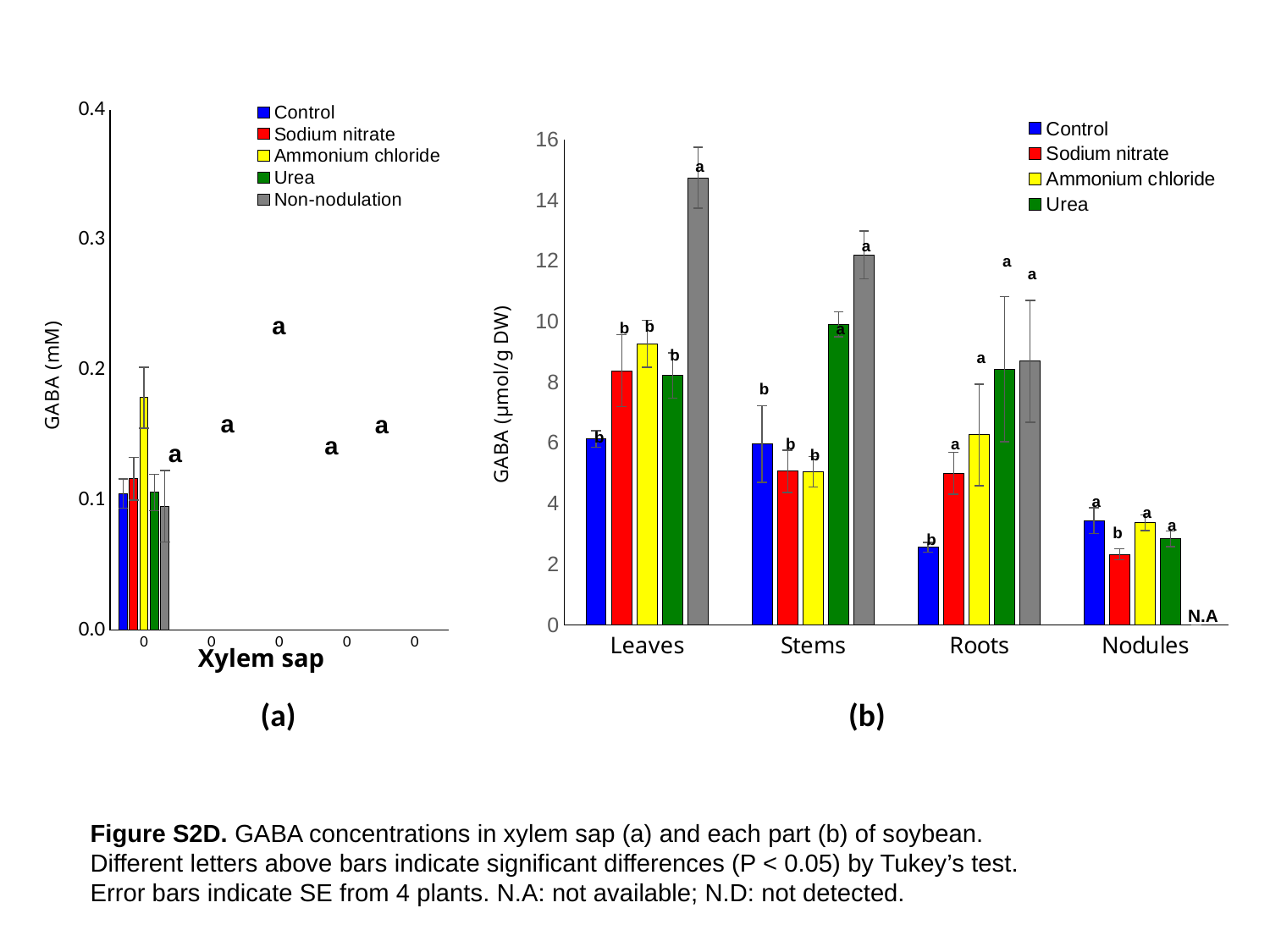

[unsupported chart]
a
a
a
a
a
### Chart
| Category | | | | | |
|---|---|---|---|---|---|
| Leaves | 6.13046218319102 | 8.37688313438375 | 9.267212529327272 | 8.21845812277665 | 14.73926230208972 |
| Stems | 5.955100896524216 | 5.060734624462548 | 5.046177476097474 | 9.909691682457243 | 12.19831926202693 |
| Roots | 2.556681636734746 | 4.996403776638663 | 6.26170595153696 | 8.42544005175695 | 8.688287647283014 |
| Nodules | 3.434617991083118 | 2.325709729437935 | 3.366007075042515 | 2.833551440766588 | 0.0 |a
a
a
a
b
b
a
b
a
b
b
b
a
b
a
a
b
a
b
N.A
Xylem sap
(a) (b)
Figure S2D. GABA concentrations in xylem sap (a) and each part (b) of soybean.
Different letters above bars indicate significant differences (P < 0.05) by Tukey’s test.
Error bars indicate SE from 4 plants. N.A: not available; N.D: not detected.

## Slide 8
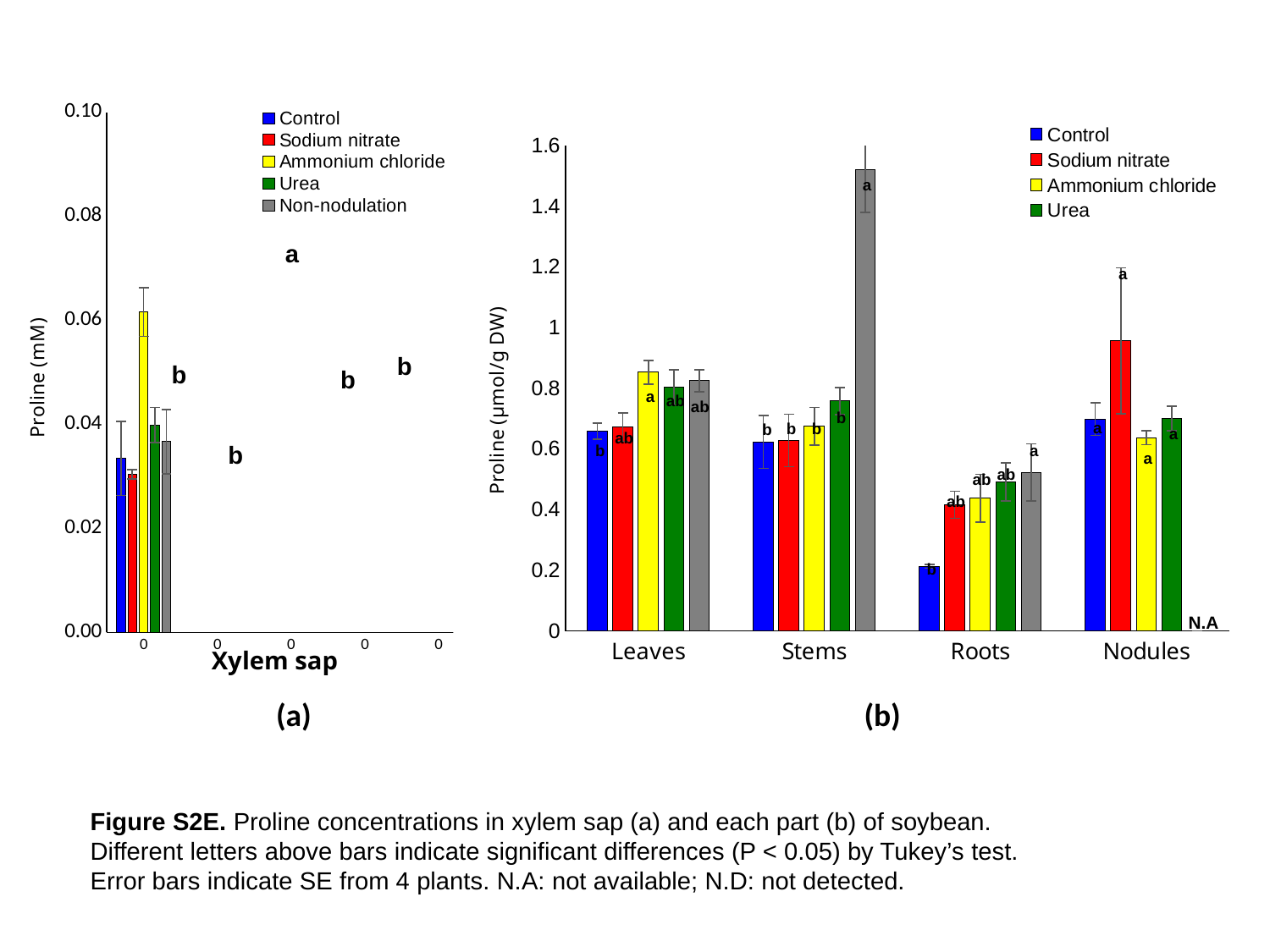

[unsupported chart]
a
b
b
b
b
### Chart
| Category | | | | | |
|---|---|---|---|---|---|
| Leaves | 0.657853630744469 | 0.670942023958261 | 0.852691815555978 | 0.802560554375388 | 0.824463356243387 |
| Stems | 0.622667067643014 | 0.628087157800946 | 0.674651013787648 | 0.757378351455391 | 1.521189758126498 |
| Roots | 0.212967772641073 | 0.415776597057883 | 0.437126974832829 | 0.490516934220716 | 0.521836194951632 |
| Nodules | 0.697762780609596 | 0.955996712292743 | 0.636323722018463 | 0.700083577314408 | 0.0 |a
a
a
ab
ab
b
a
b
b
b
a
ab
a
b
a
ab
ab
ab
b
N.A
Xylem sap
(a) (b)
Figure S2E. Proline concentrations in xylem sap (a) and each part (b) of soybean.
Different letters above bars indicate significant differences (P < 0.05) by Tukey’s test.
Error bars indicate SE from 4 plants. N.A: not available; N.D: not detected.

## Slide 9
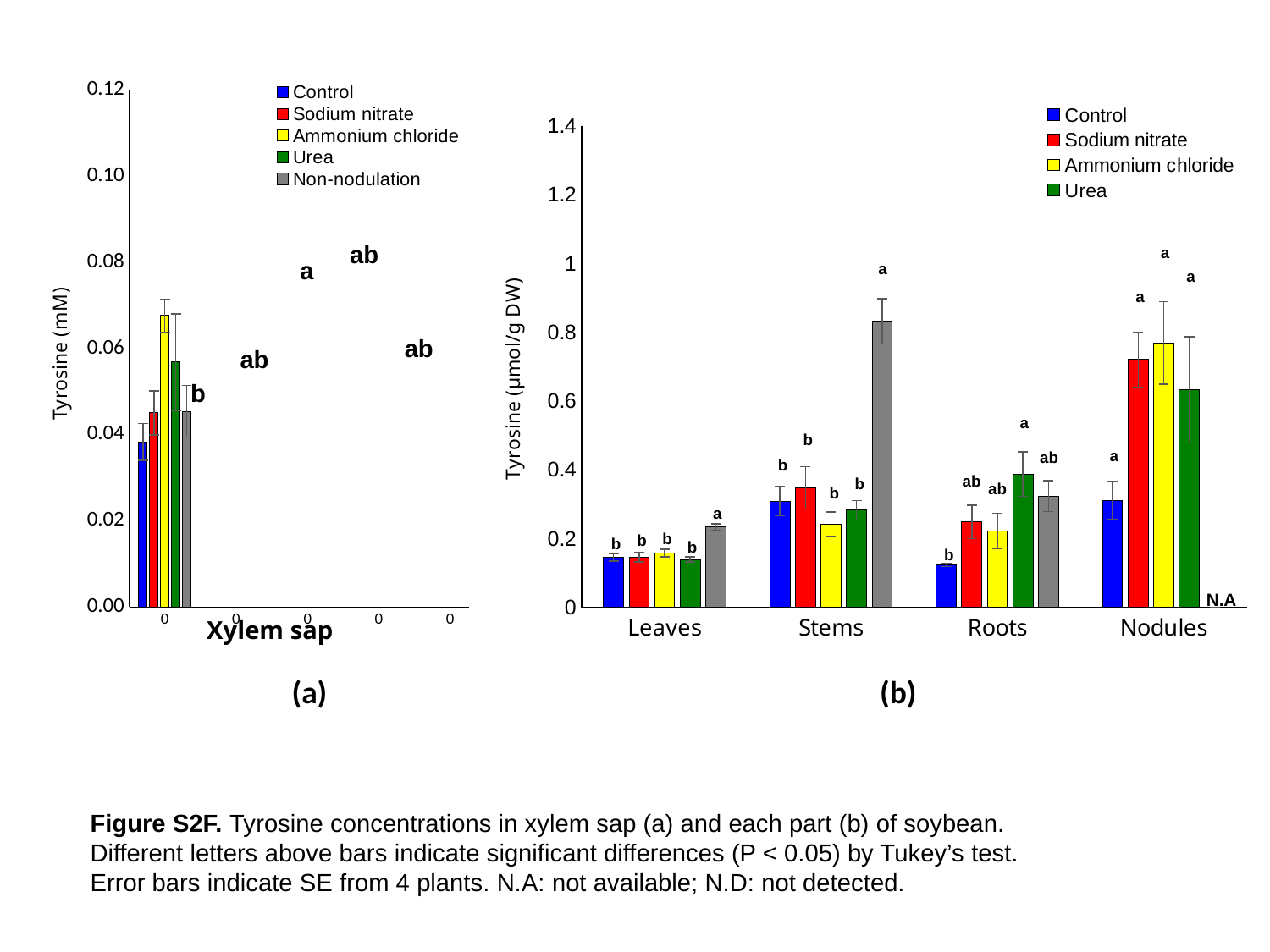

[unsupported chart]
ab
a
ab
ab
b
### Chart
| Category | | | | | |
|---|---|---|---|---|---|
| Leaves | 0.145377878618174 | 0.145454529159659 | 0.158008437310306 | 0.13948499947346 | 0.233440124148323 |
| Stems | 0.308965048916141 | 0.346976491776832 | 0.241963536507267 | 0.282463645126504 | 0.831816393497967 |
| Roots | 0.123076550554732 | 0.24835382508891 | 0.222248687658762 | 0.387958101346169 | 0.323371357238838 |
| Nodules | 0.311411573511943 | 0.720553264187195 | 0.768980842631122 | 0.633041832788763 | 0.0 |a
a
a
a
a
b
a
ab
b
ab
b
ab
b
a
b
b
b
b
b
N.A
Xylem sap
(a) (b)
Figure S2F. Tyrosine concentrations in xylem sap (a) and each part (b) of soybean.
Different letters above bars indicate significant differences (P < 0.05) by Tukey’s test.
Error bars indicate SE from 4 plants. N.A: not available; N.D: not detected.

## Slide 10
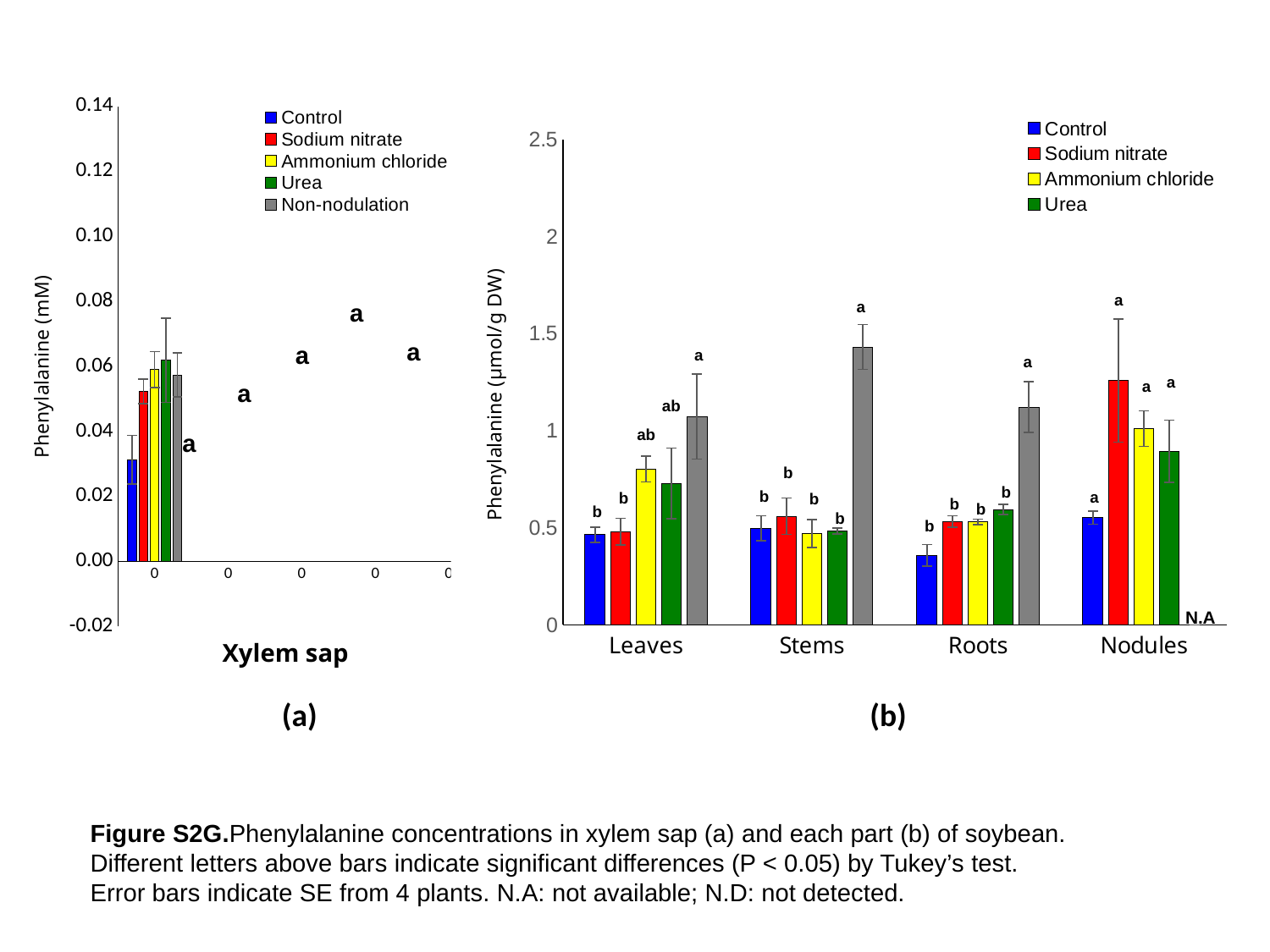

[unsupported chart]
a
a
a
a
a
### Chart
| Category | | | | | |
|---|---|---|---|---|---|
| Leaves | 0.46416443535737 | 0.480364514644264 | 0.801728757972724 | 0.728424664732318 | 1.072651743735806 |
| Stems | 0.496612317103663 | 0.558922110895578 | 0.47069643452604 | 0.48302656551912 | 1.431015383407881 |
| Roots | 0.358357931158646 | 0.53189067941737 | 0.529861048498417 | 0.5942711778508 | 1.121458240550747 |
| Nodules | 0.552304691107782 | 1.258534679726919 | 1.010299088737827 | 0.893641212393069 | 0.0 |a
a
a
a
a
a
ab
ab
b
b
b
a
b
b
b
b
b
b
b
N.A
Xylem sap
(a) (b)
Figure S2G.Phenylalanine concentrations in xylem sap (a) and each part (b) of soybean.
Different letters above bars indicate significant differences (P < 0.05) by Tukey’s test.
Error bars indicate SE from 4 plants. N.A: not available; N.D: not detected.

## Slide 11
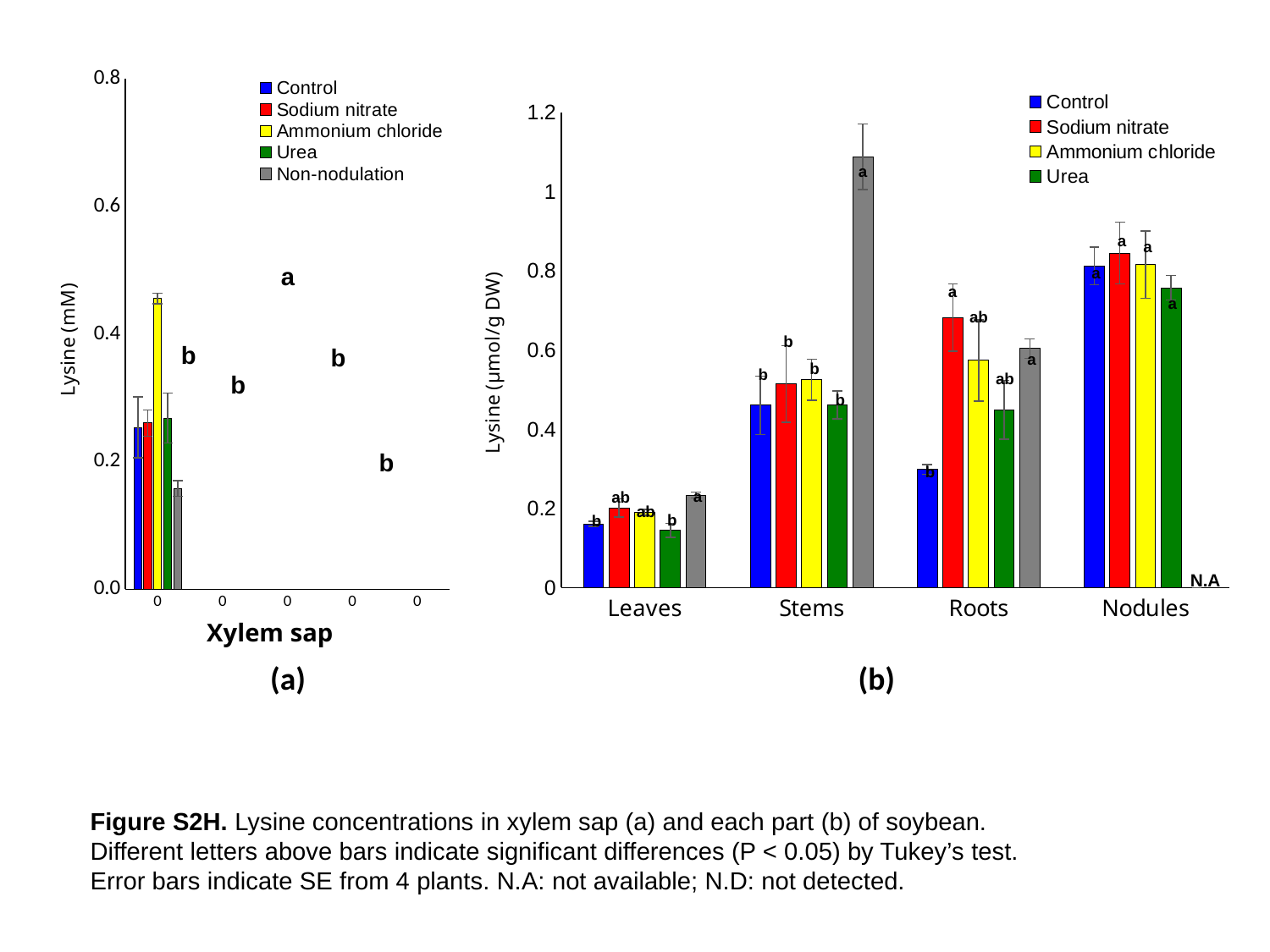

[unsupported chart]
a
b
b
b
b
### Chart
| Category | | | | | |
|---|---|---|---|---|---|
| Leaves | 0.161143365223816 | 0.201046394826739 | 0.190155084565361 | 0.144483291341163 | 0.233570982383148 |
| Stems | 0.460700606855423 | 0.514438035730614 | 0.525601858387517 | 0.461879684470172 | 1.089445373246038 |
| Roots | 0.298985488141187 | 0.682699684288355 | 0.574131412658204 | 0.449557380714054 | 0.604067111891599 |
| Nodules | 0.81302175339672 | 0.845620037485385 | 0.815899004501107 | 0.757895269807224 | 0.0 |a
a
a
a
a
ab
b
a
b
b
ab
b
b
a
ab
ab
b
b
N.A
a
Xylem sap
(a) (b)
Figure S2H. Lysine concentrations in xylem sap (a) and each part (b) of soybean.
Different letters above bars indicate significant differences (P < 0.05) by Tukey’s test.
Error bars indicate SE from 4 plants. N.A: not available; N.D: not detected.

## Slide 12
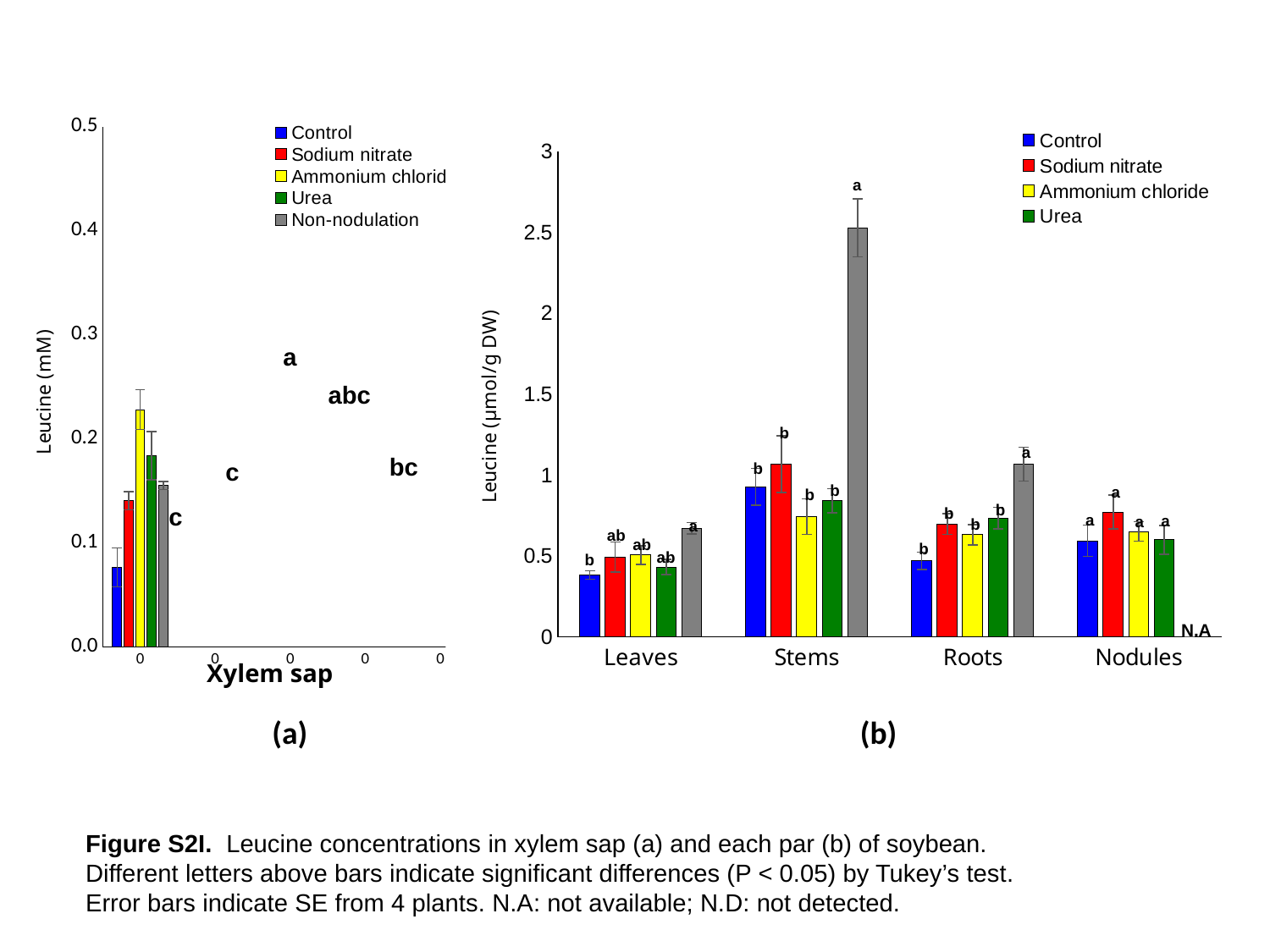

[unsupported chart]
a
abc
bc
c
c
### Chart
| Category | | | | | |
|---|---|---|---|---|---|
| Leaves | 0.381083754915839 | 0.49326957330926 | 0.505434350666245 | 0.431084686451873 | 0.671150824710536 |
| Stems | 0.927248500111344 | 1.06699158810953 | 0.74252282346415 | 0.840938369207294 | 2.527986083495015 |
| Roots | 0.470250931473196 | 0.696379357677475 | 0.631115570692038 | 0.733518451297185 | 1.067134758382736 |
| Nodules | 0.59336806084221 | 0.771476483317414 | 0.651064129279781 | 0.599711960657543 | 0.0 |a
b
a
b
b
a
b
b
b
a
a
a
b
a
ab
ab
b
b
ab
N.A
Xylem sap
(a) (b)
Figure S2I. Leucine concentrations in xylem sap (a) and each par (b) of soybean.
Different letters above bars indicate significant differences (P < 0.05) by Tukey’s test.
Error bars indicate SE from 4 plants. N.A: not available; N.D: not detected.

## Slide 13
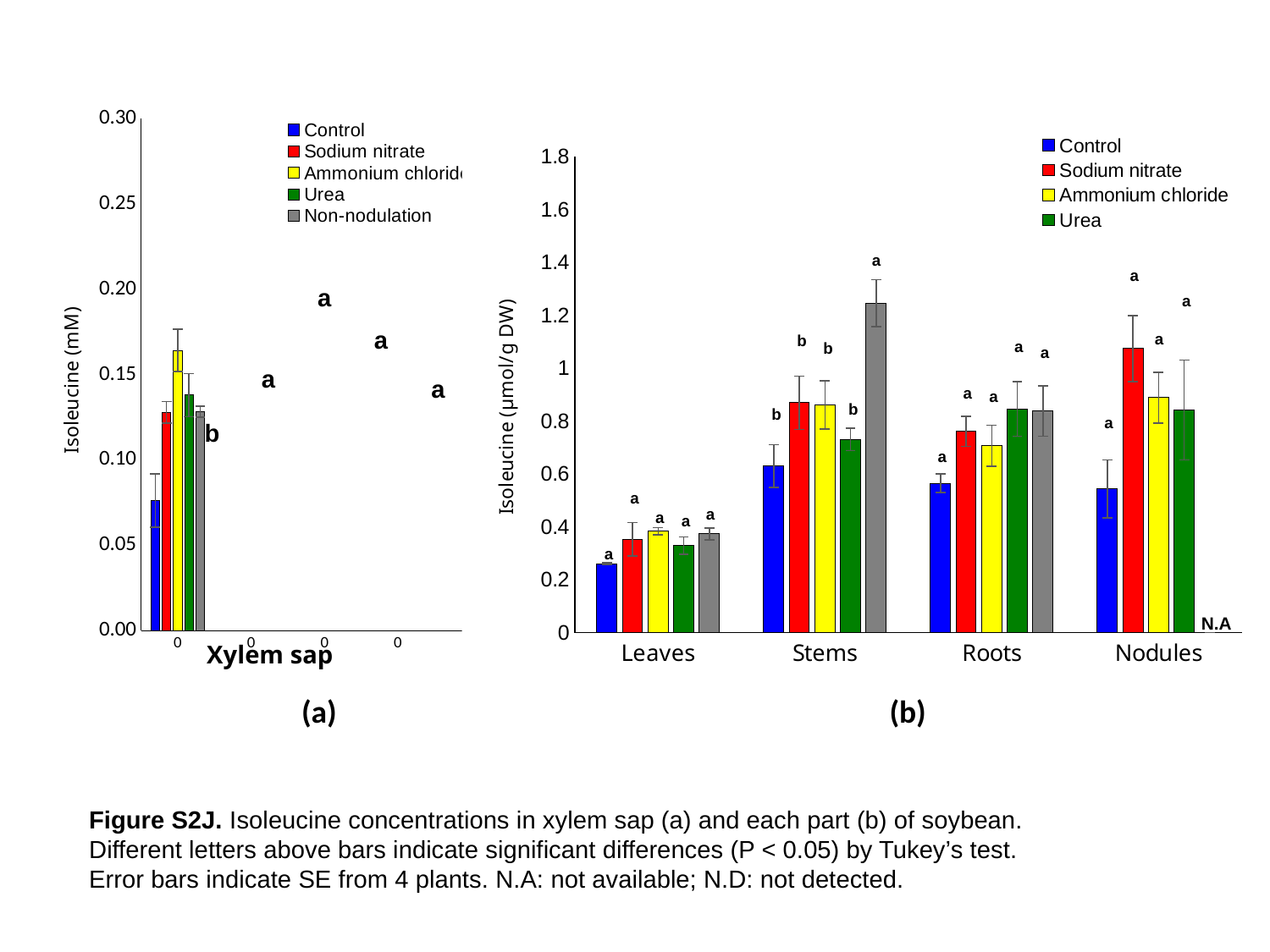

[unsupported chart]
a
a
a
a
b
### Chart
| Category | | | | | |
|---|---|---|---|---|---|
| Leaves | 0.260255637477231 | 0.352879230562186 | 0.382905234485567 | 0.329624881728433 | 0.373309170962774 |
| Stems | 0.629575889973865 | 0.868940565374927 | 0.860777375975668 | 0.730270552773789 | 1.245284400373247 |
| Roots | 0.564715135443683 | 0.761449057662503 | 0.706473197953768 | 0.84557458613296 | 0.837448395475933 |
| Nodules | 0.543349581967197 | 1.073902505258371 | 0.888190745555333 | 0.841815495869193 | 0.0 |a
a
a
a
b
a
b
a
a
a
b
b
a
a
a
a
a
a
a
N.A
Xylem sap
(a) (b)
Figure S2J. Isoleucine concentrations in xylem sap (a) and each part (b) of soybean.
Different letters above bars indicate significant differences (P < 0.05) by Tukey’s test.
Error bars indicate SE from 4 plants. N.A: not available; N.D: not detected.

## Slide 14
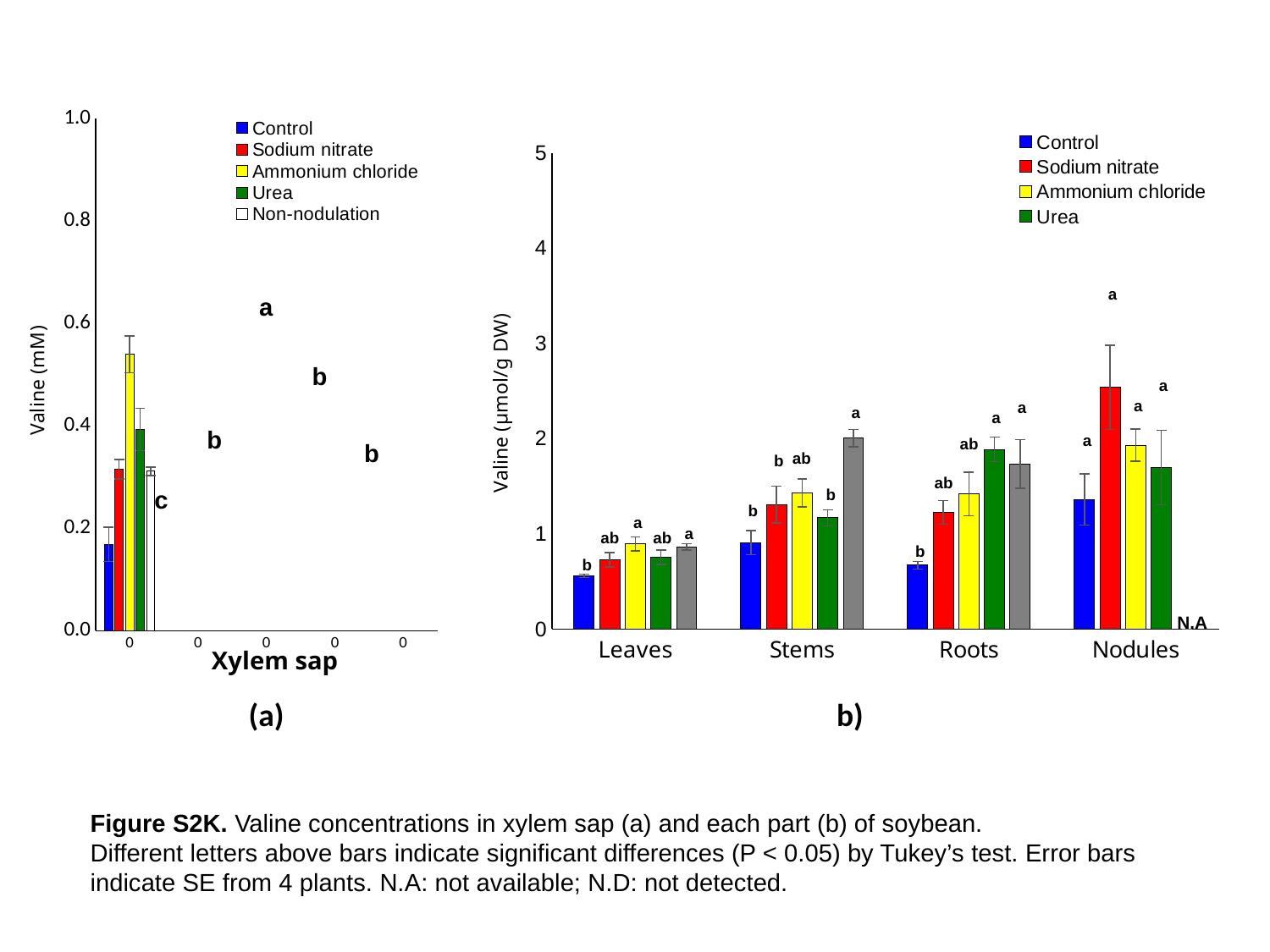

[unsupported chart]
a
b
b
b
c
### Chart
| Category | | | | | |
|---|---|---|---|---|---|
| Leaves | 0.560347915614968 | 0.727939502142318 | 0.894187847418232 | 0.753591131827523 | 0.862519386262761 |
| Stems | 0.908545916826439 | 1.307132479964331 | 1.428692043783345 | 1.168223606073338 | 2.003675966830285 |
| Roots | 0.671651277802936 | 1.225524280198169 | 1.418017448945283 | 1.885116072183015 | 1.733844958283804 |
| Nodules | 1.36060280624727 | 2.536547916407399 | 1.929709878984497 | 1.695436778483843 | 0.0 |a
a
a
a
a
a
a
ab
ab
b
ab
b
b
a
a
ab
ab
b
b
N.A
Xylem sap
(a) b)
Figure S2K. Valine concentrations in xylem sap (a) and each part (b) of soybean.
Different letters above bars indicate significant differences (P < 0.05) by Tukey’s test. Error bars indicate SE from 4 plants. N.A: not available; N.D: not detected.

## Slide 15
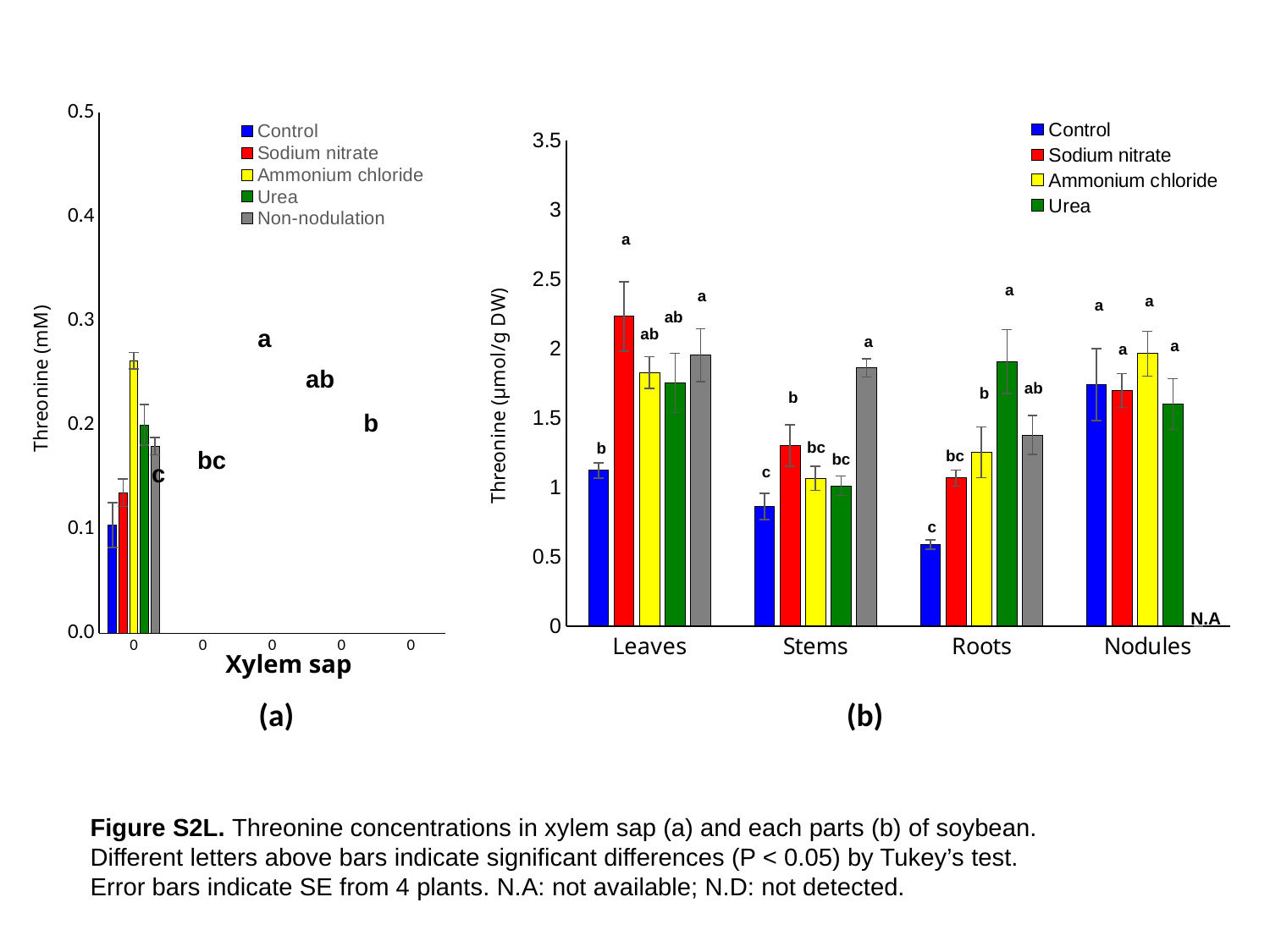

[unsupported chart]
a
ab
b
bc
c
### Chart
| Category | | | | | |
|---|---|---|---|---|---|
| Leaves | 1.122155234667941 | 2.232266815394043 | 1.829156787119668 | 1.753252850202286 | 1.953034951356127 |
| Stems | 0.861372715687797 | 1.301458457990454 | 1.064605037129168 | 1.010773884631853 | 1.86199124424304 |
| Roots | 0.588299052175266 | 1.066913216195979 | 1.253270343959841 | 1.90667812032839 | 1.37752343659531 |
| Nodules | 1.741899023177091 | 1.69865880329056 | 1.964103386686088 | 1.601348998720048 | 0.0 |a
a
a
a
a
ab
ab
a
a
a
ab
b
b
b
bc
bc
bc
c
c
N.A
Xylem sap
(a) (b)
Figure S2L. Threonine concentrations in xylem sap (a) and each parts (b) of soybean.
Different letters above bars indicate significant differences (P < 0.05) by Tukey’s test.
Error bars indicate SE from 4 plants. N.A: not available; N.D: not detected.

## Slide 16
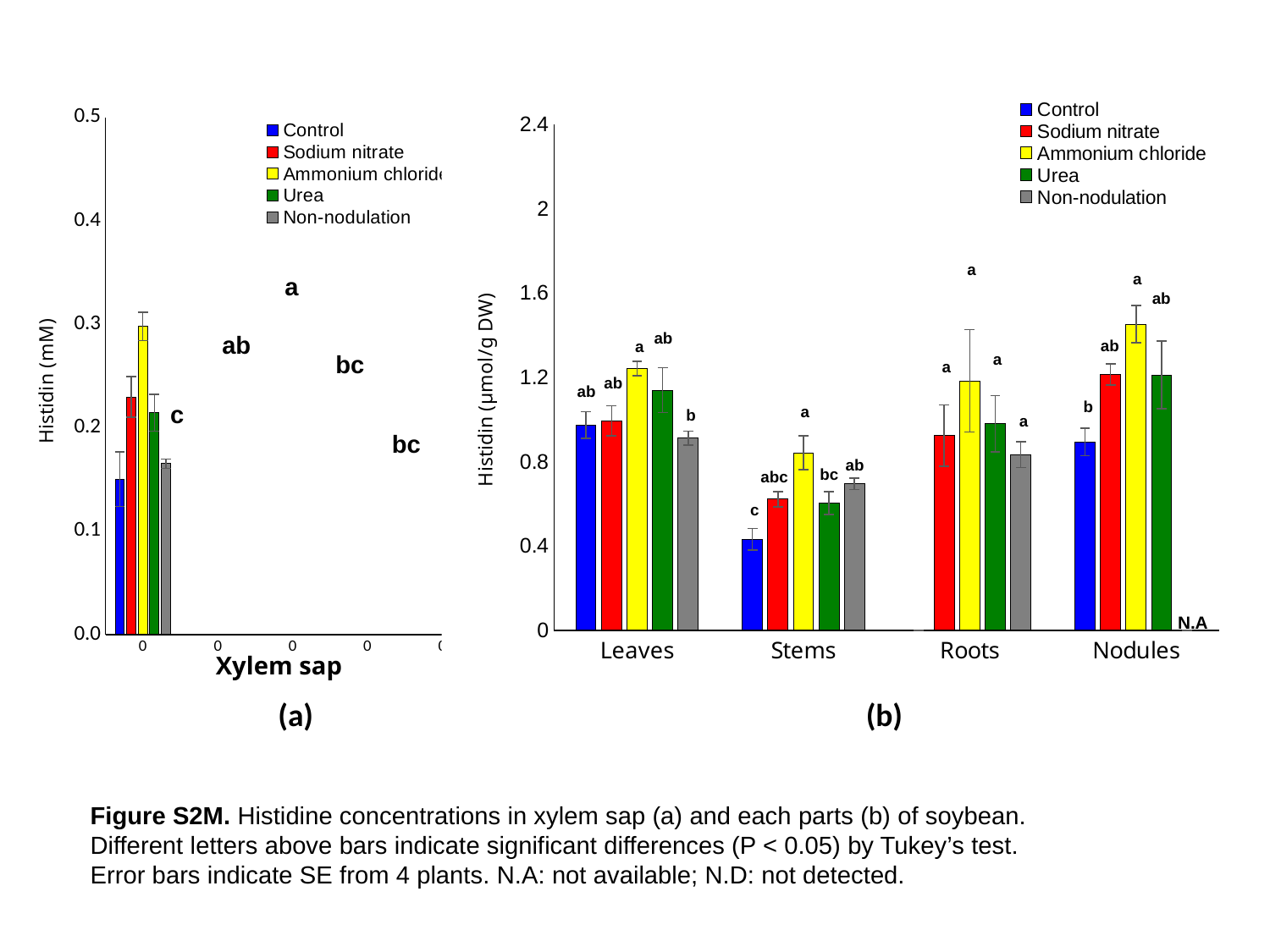

[unsupported chart]
a
ab
bc
c
bc
### Chart
| Category | | | | | |
|---|---|---|---|---|---|
| Leaves | 0.973969011370276 | 0.993998918173753 | 1.241033291312256 | 1.13857997289175 | 0.910696123152244 |
| Stems | 0.431848057530976 | 0.621806937253637 | 0.8418924747379 | 0.603660945726835 | 0.69538461334635 |
| Roots | 0.0 | 0.923795655752491 | 1.1835890355418 | 0.979365647130691 | 0.832853161041048 |
| Nodules | 0.89377604280266 | 1.21284239674316 | 1.452466270006 | 1.211359131936367 | 0.0 |a
a
ab
ab
ab
a
a
a
ab
ab
b
a
b
a
ab
bc
abc
c
N.A
Xylem sap
(a) (b)
Figure S2M. Histidine concentrations in xylem sap (a) and each parts (b) of soybean.
Different letters above bars indicate significant differences (P < 0.05) by Tukey’s test.
Error bars indicate SE from 4 plants. N.A: not available; N.D: not detected.

## Slide 17
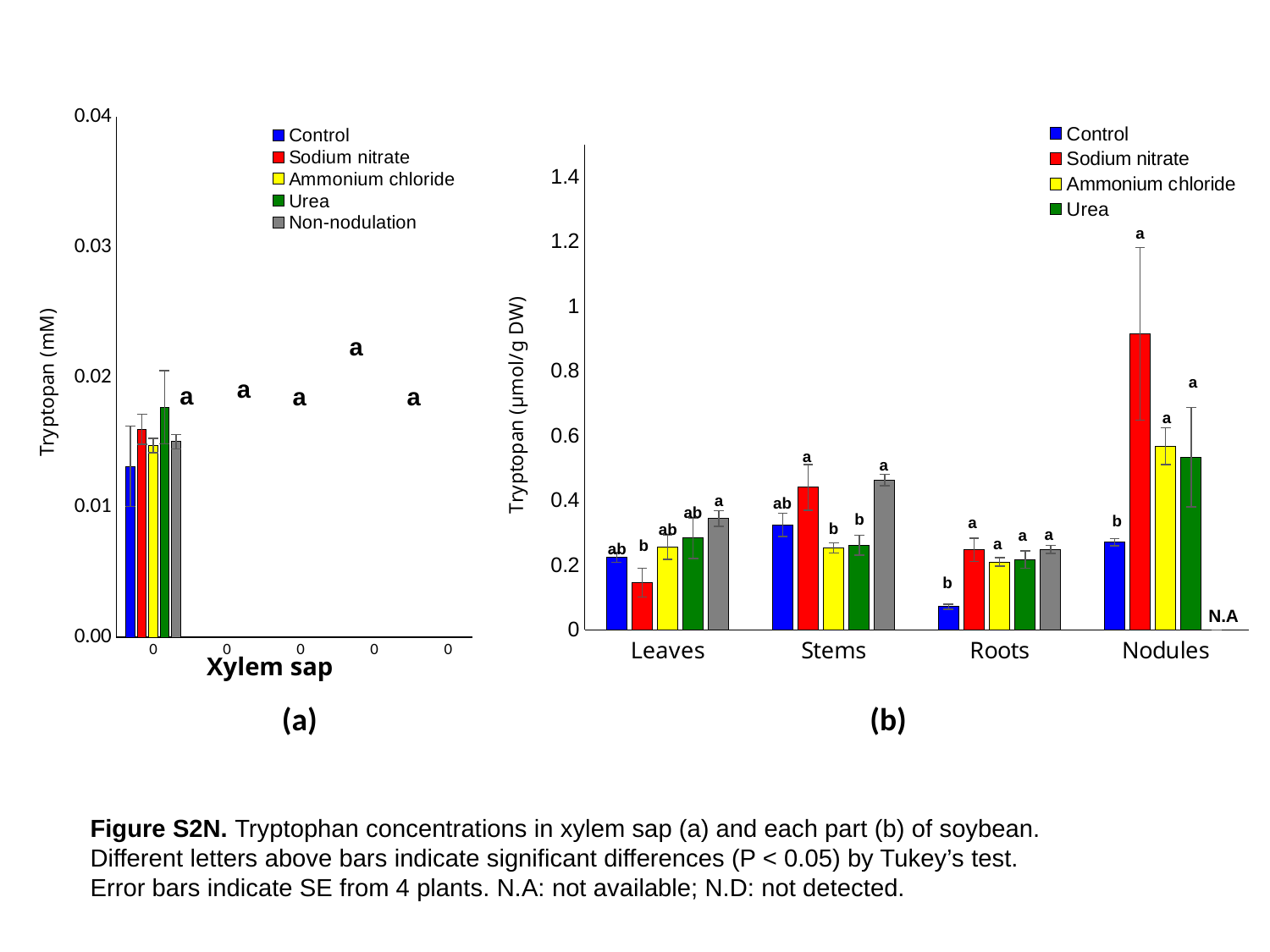

[unsupported chart]
a
a
a
a
a
### Chart
| Category | | | | | |
|---|---|---|---|---|---|
| Leaves | 0.222961961324832 | 0.145885411686517 | 0.256174074103718 | 0.283615528705692 | 0.343487167689574 |
| Stems | 0.324154280163833 | 0.439976188117664 | 0.253250783485932 | 0.26129012990394 | 0.462988672962019 |
| Roots | 0.0717610096132673 | 0.247266024850149 | 0.209797543233269 | 0.216944962488279 | 0.248634091077411 |
| Nodules | 0.270683092665955 | 0.914473551472215 | 0.567977724284969 | 0.533756802259407 | 0.0 |a
a
a
a
a
a
ab
ab
b
b
a
b
ab
a
a
a
b
ab
b
N.A
Xylem sap
(a) (b)
Figure S2N. Tryptophan concentrations in xylem sap (a) and each part (b) of soybean.
Different letters above bars indicate significant differences (P < 0.05) by Tukey’s test.
Error bars indicate SE from 4 plants. N.A: not available; N.D: not detected.

## Slide 18
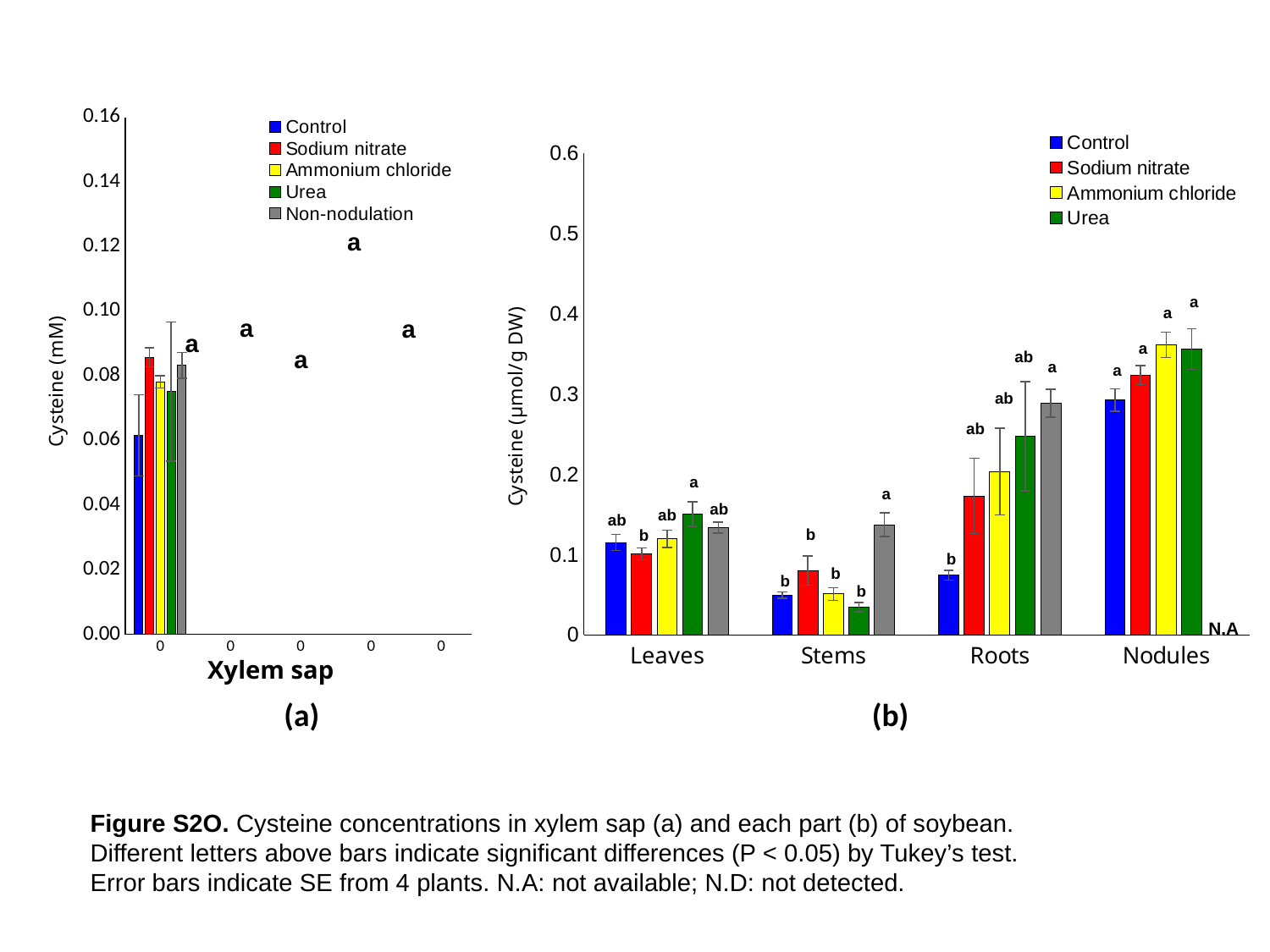

[unsupported chart]
a
a
a
a
a
### Chart
| Category | | | | | |
|---|---|---|---|---|---|
| Leaves | 0.115210732991511 | 0.101074898267883 | 0.119887311168368 | 0.150858167058723 | 0.133725487188157 |
| Stems | 0.0497977126004913 | 0.0801844338935027 | 0.0511017390410546 | 0.0343526454601698 | 0.137279900455276 |
| Roots | 0.0743562375918194 | 0.173158156203234 | 0.203655037736219 | 0.247621416847644 | 0.288935688117582 |
| Nodules | 0.292758478732914 | 0.323985475196758 | 0.36177706543454 | 0.356277539247749 | 0.0 |a
a
a
ab
a
a
ab
ab
a
a
ab
ab
ab
b
b
b
b
b
b
N.A
Xylem sap
(a) (b)
Figure S2O. Cysteine concentrations in xylem sap (a) and each part (b) of soybean.
Different letters above bars indicate significant differences (P < 0.05) by Tukey’s test.
Error bars indicate SE from 4 plants. N.A: not available; N.D: not detected.

## Slide 19
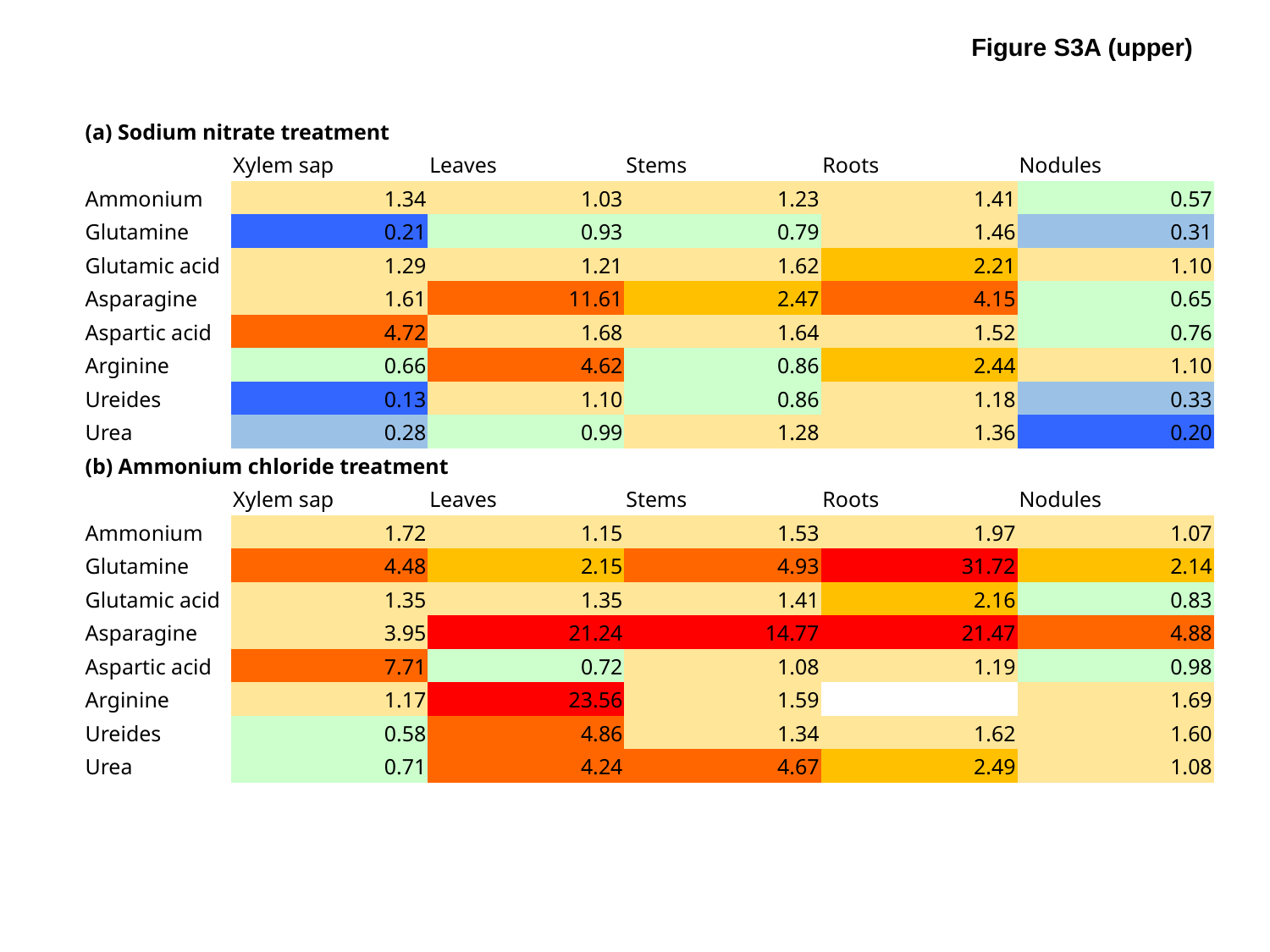

Figure S3A (upper)
| | | | | | |
| --- | --- | --- | --- | --- | --- |
| | | | | | |
| (a) Sodium nitrate treatment | | | | | |
| | Xylem sap | Leaves | Stems | Roots | Nodules |
| Ammonium | 1.34 | 1.03 | 1.23 | 1.41 | 0.57 |
| Glutamine | 0.21 | 0.93 | 0.79 | 1.46 | 0.31 |
| Glutamic acid | 1.29 | 1.21 | 1.62 | 2.21 | 1.10 |
| Asparagine | 1.61 | 11.61 | 2.47 | 4.15 | 0.65 |
| Aspartic acid | 4.72 | 1.68 | 1.64 | 1.52 | 0.76 |
| Arginine | 0.66 | 4.62 | 0.86 | 2.44 | 1.10 |
| Ureides | 0.13 | 1.10 | 0.86 | 1.18 | 0.33 |
| Urea | 0.28 | 0.99 | 1.28 | 1.36 | 0.20 |
| (b) Ammonium chloride treatment | | | | | |
| | Xylem sap | Leaves | Stems | Roots | Nodules |
| Ammonium | 1.72 | 1.15 | 1.53 | 1.97 | 1.07 |
| Glutamine | 4.48 | 2.15 | 4.93 | 31.72 | 2.14 |
| Glutamic acid | 1.35 | 1.35 | 1.41 | 2.16 | 0.83 |
| Asparagine | 3.95 | 21.24 | 14.77 | 21.47 | 4.88 |
| Aspartic acid | 7.71 | 0.72 | 1.08 | 1.19 | 0.98 |
| Arginine | 1.17 | 23.56 | 1.59 | | 1.69 |
| Ureides | 0.58 | 4.86 | 1.34 | 1.62 | 1.60 |
| Urea | 0.71 | 4.24 | 4.67 | 2.49 | 1.08 |

## Slide 20
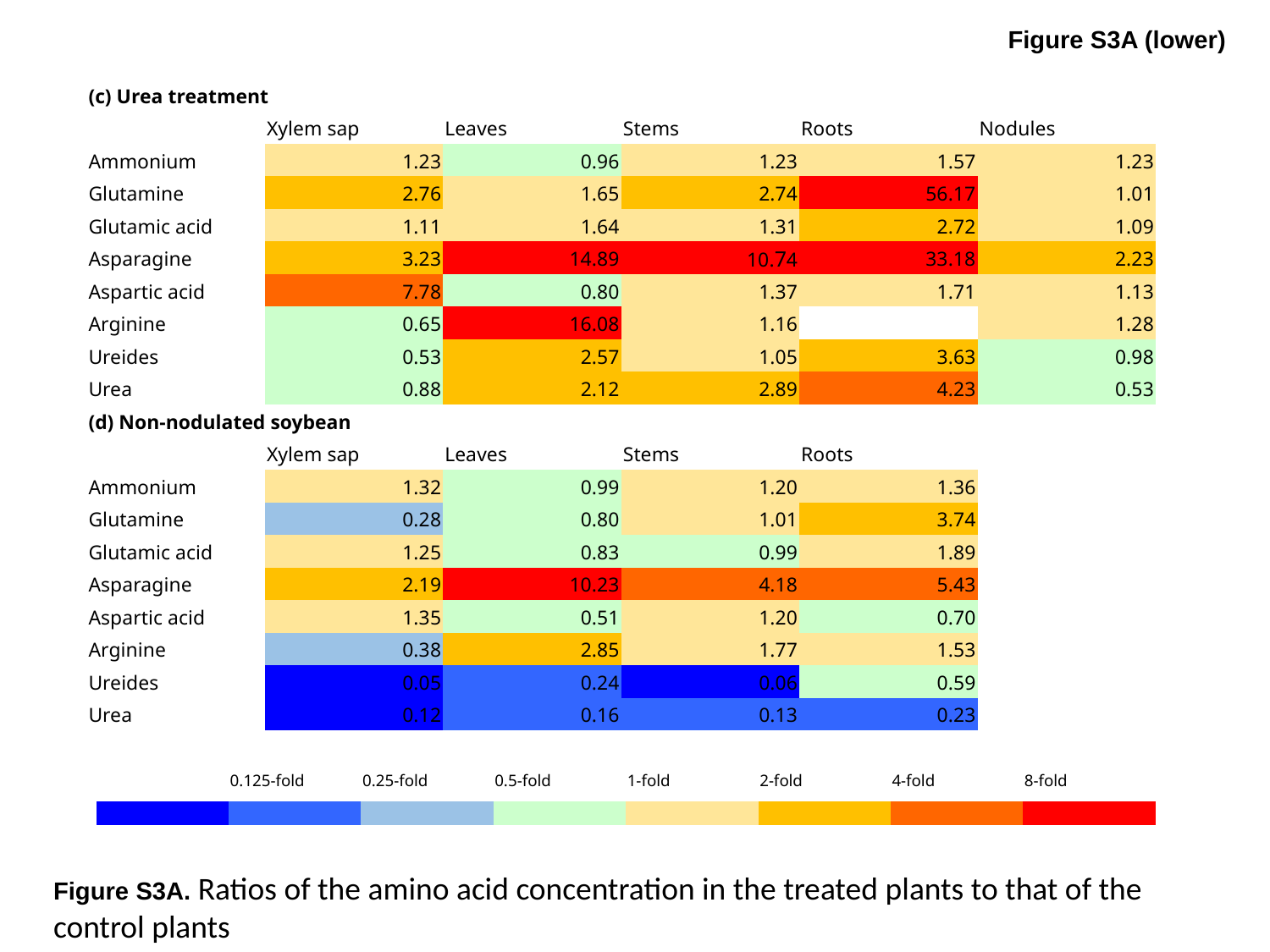

Figure S3A (lower)
| (c) Urea treatment | | | | | |
| --- | --- | --- | --- | --- | --- |
| | Xylem sap | Leaves | Stems | Roots | Nodules |
| Ammonium | 1.23 | 0.96 | 1.23 | 1.57 | 1.23 |
| Glutamine | 2.76 | 1.65 | 2.74 | 56.17 | 1.01 |
| Glutamic acid | 1.11 | 1.64 | 1.31 | 2.72 | 1.09 |
| Asparagine | 3.23 | 14.89 | 10.74 | 33.18 | 2.23 |
| Aspartic acid | 7.78 | 0.80 | 1.37 | 1.71 | 1.13 |
| Arginine | 0.65 | 16.08 | 1.16 | | 1.28 |
| Ureides | 0.53 | 2.57 | 1.05 | 3.63 | 0.98 |
| Urea | 0.88 | 2.12 | 2.89 | 4.23 | 0.53 |
| (d) Non-nodulated soybean | | | | | |
| | Xylem sap | Leaves | Stems | Roots | |
| Ammonium | 1.32 | 0.99 | 1.20 | 1.36 | |
| Glutamine | 0.28 | 0.80 | 1.01 | 3.74 | |
| Glutamic acid | 1.25 | 0.83 | 0.99 | 1.89 | |
| Asparagine | 2.19 | 10.23 | 4.18 | 5.43 | |
| Aspartic acid | 1.35 | 0.51 | 1.20 | 0.70 | |
| Arginine | 0.38 | 2.85 | 1.77 | 1.53 | |
| Ureides | 0.05 | 0.24 | 0.06 | 0.59 | |
| Urea | 0.12 | 0.16 | 0.13 | 0.23 | |
| | 0.125-fold | 0.25-fold | 0.5-fold | 1-fold | 2-fold | 4-fold | 8-fold |
| --- | --- | --- | --- | --- | --- | --- | --- |
| | | | | | | | |
Figure S3A. Ratios of the amino acid concentration in the treated plants to that of the control plants

## Slide 21
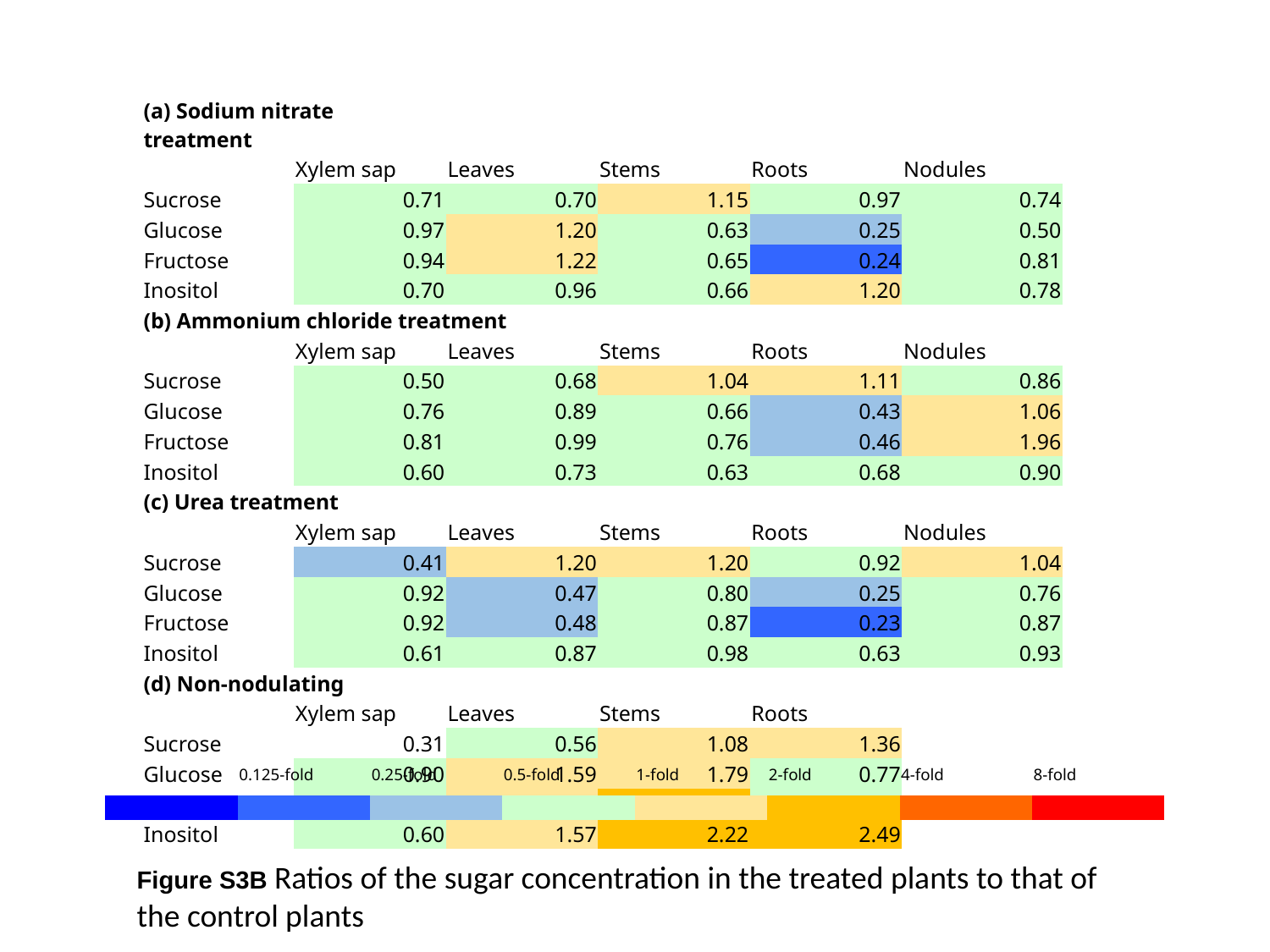

| | | | | | |
| --- | --- | --- | --- | --- | --- |
| | | | | | |
| (a) Sodium nitrate treatment | | | | | |
| | Xylem sap | Leaves | Stems | Roots | Nodules |
| Sucrose | 0.71 | 0.70 | 1.15 | 0.97 | 0.74 |
| Glucose | 0.97 | 1.20 | 0.63 | 0.25 | 0.50 |
| Fructose | 0.94 | 1.22 | 0.65 | 0.24 | 0.81 |
| Inositol | 0.70 | 0.96 | 0.66 | 1.20 | 0.78 |
| (b) Ammonium chloride treatment | | | | | |
| | Xylem sap | Leaves | Stems | Roots | Nodules |
| Sucrose | 0.50 | 0.68 | 1.04 | 1.11 | 0.86 |
| Glucose | 0.76 | 0.89 | 0.66 | 0.43 | 1.06 |
| Fructose | 0.81 | 0.99 | 0.76 | 0.46 | 1.96 |
| Inositol | 0.60 | 0.73 | 0.63 | 0.68 | 0.90 |
| (c) Urea treatment | | | | | |
| | Xylem sap | Leaves | Stems | Roots | Nodules |
| Sucrose | 0.41 | 1.20 | 1.20 | 0.92 | 1.04 |
| Glucose | 0.92 | 0.47 | 0.80 | 0.25 | 0.76 |
| Fructose | 0.92 | 0.48 | 0.87 | 0.23 | 0.87 |
| Inositol | 0.61 | 0.87 | 0.98 | 0.63 | 0.93 |
| (d) Non-nodulating | | | | | |
| | Xylem sap | Leaves | Stems | Roots | |
| Sucrose | 0.31 | 0.56 | 1.08 | 1.36 | |
| Glucose | 0.90 | 1.59 | 1.79 | 0.77 | |
| Fructose | 0.97 | 1.93 | 2.05 | 0.61 | |
| Inositol | 0.60 | 1.57 | 2.22 | 2.49 | |
| | 0.125-fold | 0.25-fold | 0.5-fold | 1-fold | 2-fold | 4-fold | 8-fold |
| --- | --- | --- | --- | --- | --- | --- | --- |
| | | | | | | | |
Figure S3B Ratios of the sugar concentration in the treated plants to that of the control plants

## Slide 22
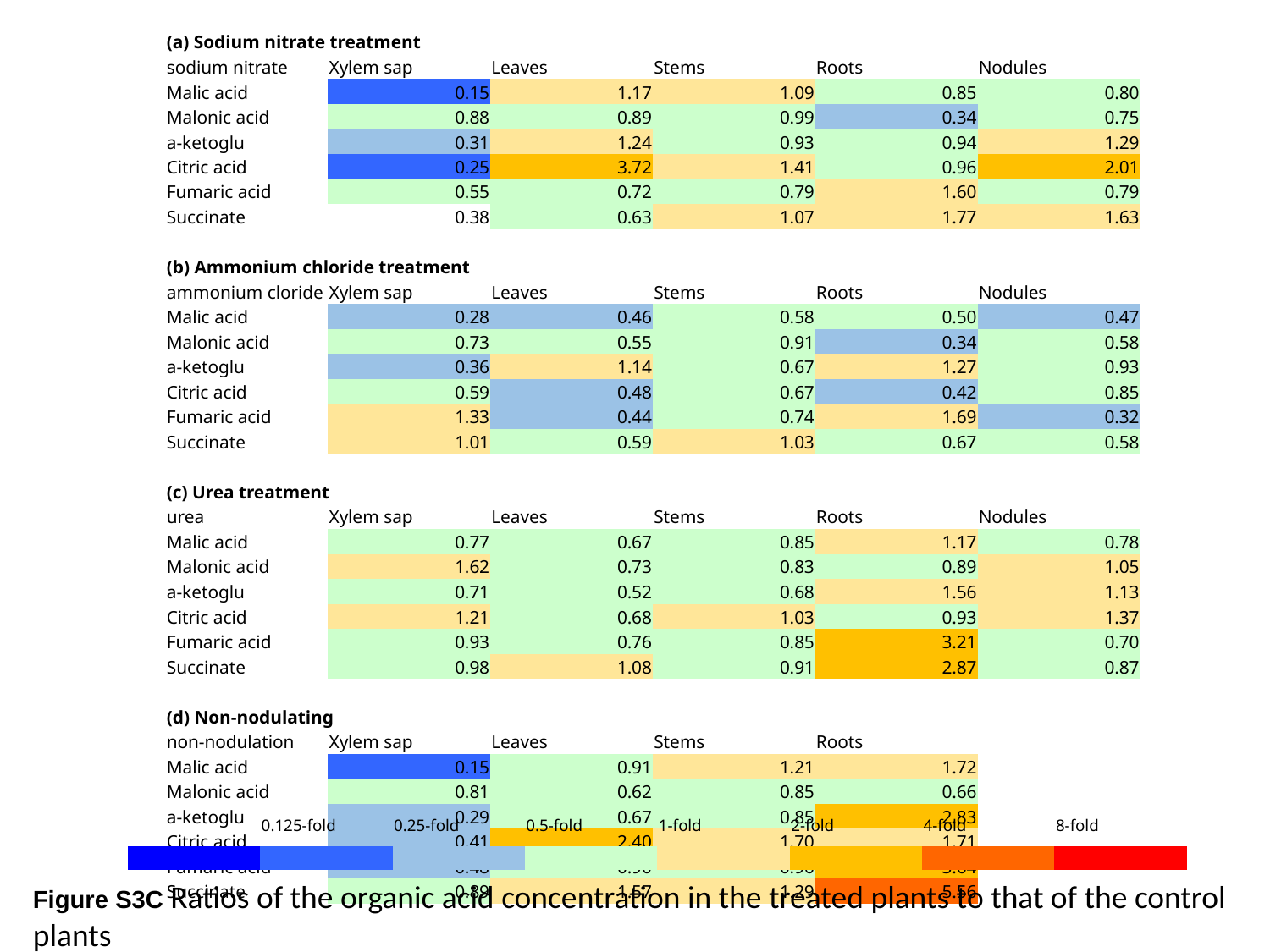

| | | | | | |
| --- | --- | --- | --- | --- | --- |
| (a) Sodium nitrate treatment | | | | | |
| sodium nitrate | Xylem sap | Leaves | Stems | Roots | Nodules |
| Malic acid | 0.15 | 1.17 | 1.09 | 0.85 | 0.80 |
| Malonic acid | 0.88 | 0.89 | 0.99 | 0.34 | 0.75 |
| a-ketoglu | 0.31 | 1.24 | 0.93 | 0.94 | 1.29 |
| Citric acid | 0.25 | 3.72 | 1.41 | 0.96 | 2.01 |
| Fumaric acid | 0.55 | 0.72 | 0.79 | 1.60 | 0.79 |
| Succinate | 0.38 | 0.63 | 1.07 | 1.77 | 1.63 |
| | | | | | |
| (b) Ammonium chloride treatment | | | | | |
| ammonium cloride | Xylem sap | Leaves | Stems | Roots | Nodules |
| Malic acid | 0.28 | 0.46 | 0.58 | 0.50 | 0.47 |
| Malonic acid | 0.73 | 0.55 | 0.91 | 0.34 | 0.58 |
| a-ketoglu | 0.36 | 1.14 | 0.67 | 1.27 | 0.93 |
| Citric acid | 0.59 | 0.48 | 0.67 | 0.42 | 0.85 |
| Fumaric acid | 1.33 | 0.44 | 0.74 | 1.69 | 0.32 |
| Succinate | 1.01 | 0.59 | 1.03 | 0.67 | 0.58 |
| | | | | | |
| (c) Urea treatment | | | | | |
| urea | Xylem sap | Leaves | Stems | Roots | Nodules |
| Malic acid | 0.77 | 0.67 | 0.85 | 1.17 | 0.78 |
| Malonic acid | 1.62 | 0.73 | 0.83 | 0.89 | 1.05 |
| a-ketoglu | 0.71 | 0.52 | 0.68 | 1.56 | 1.13 |
| Citric acid | 1.21 | 0.68 | 1.03 | 0.93 | 1.37 |
| Fumaric acid | 0.93 | 0.76 | 0.85 | 3.21 | 0.70 |
| Succinate | 0.98 | 1.08 | 0.91 | 2.87 | 0.87 |
| | | | | | |
| (d) Non-nodulating | | | | | |
| non-nodulation | Xylem sap | Leaves | Stems | Roots | |
| Malic acid | 0.15 | 0.91 | 1.21 | 1.72 | |
| Malonic acid | 0.81 | 0.62 | 0.85 | 0.66 | |
| a-ketoglu | 0.29 | 0.67 | 0.85 | 2.83 | |
| Citric acid | 0.41 | 2.40 | 1.70 | 1.71 | |
| Fumaric acid | 0.48 | 0.90 | 0.96 | 3.64 | |
| Succinate | 0.89 | 1.57 | 1.29 | 5.56 | |
| | 0.125-fold | 0.25-fold | 0.5-fold | 1-fold | 2-fold | 4-fold | 8-fold |
| --- | --- | --- | --- | --- | --- | --- | --- |
| | | | | | | | |
Figure S3C Ratios of the organic acid concentration in the treated plants to that of the control plants

## Slide 23
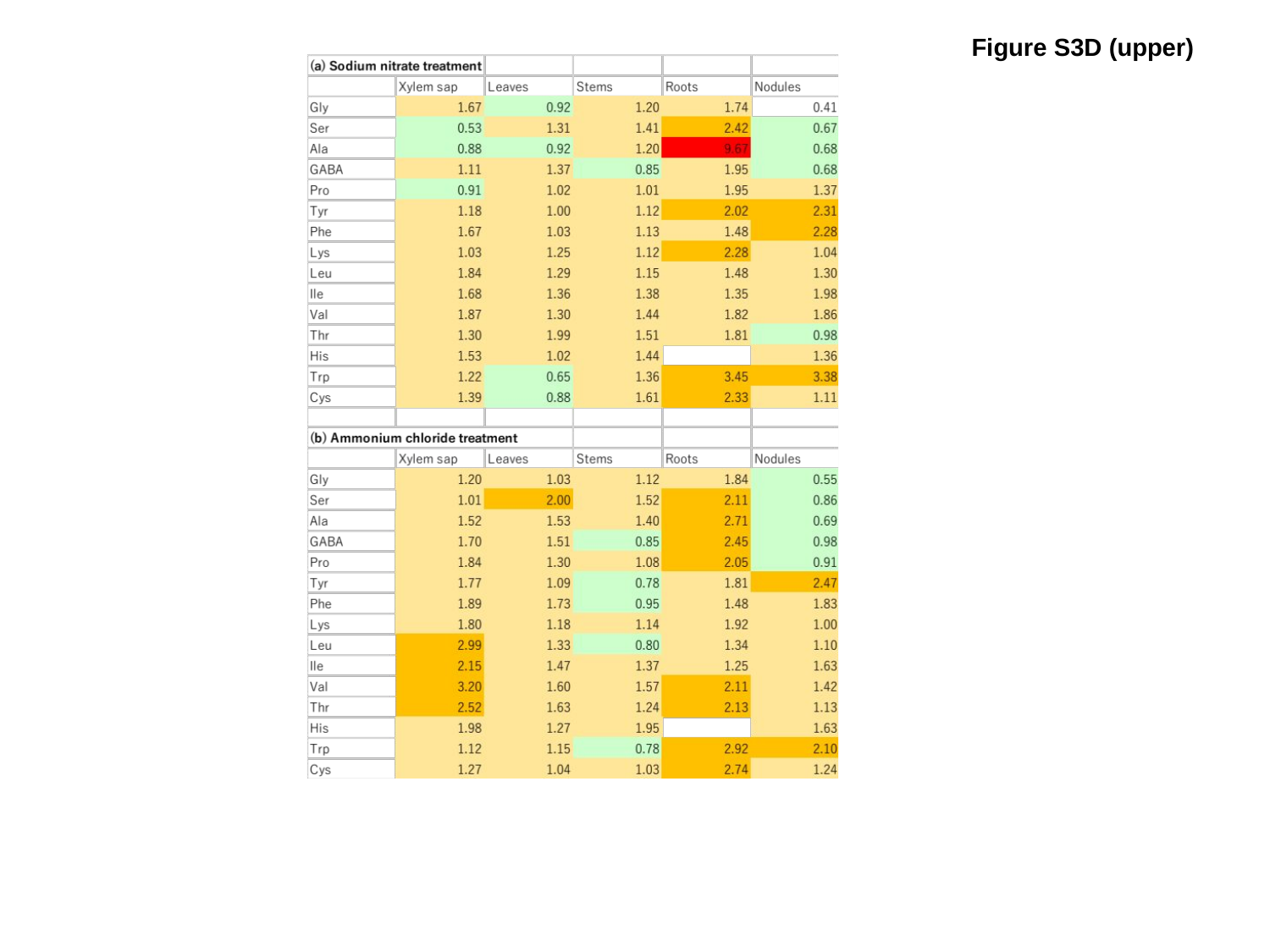

Figure S3D (upper)

## Slide 24
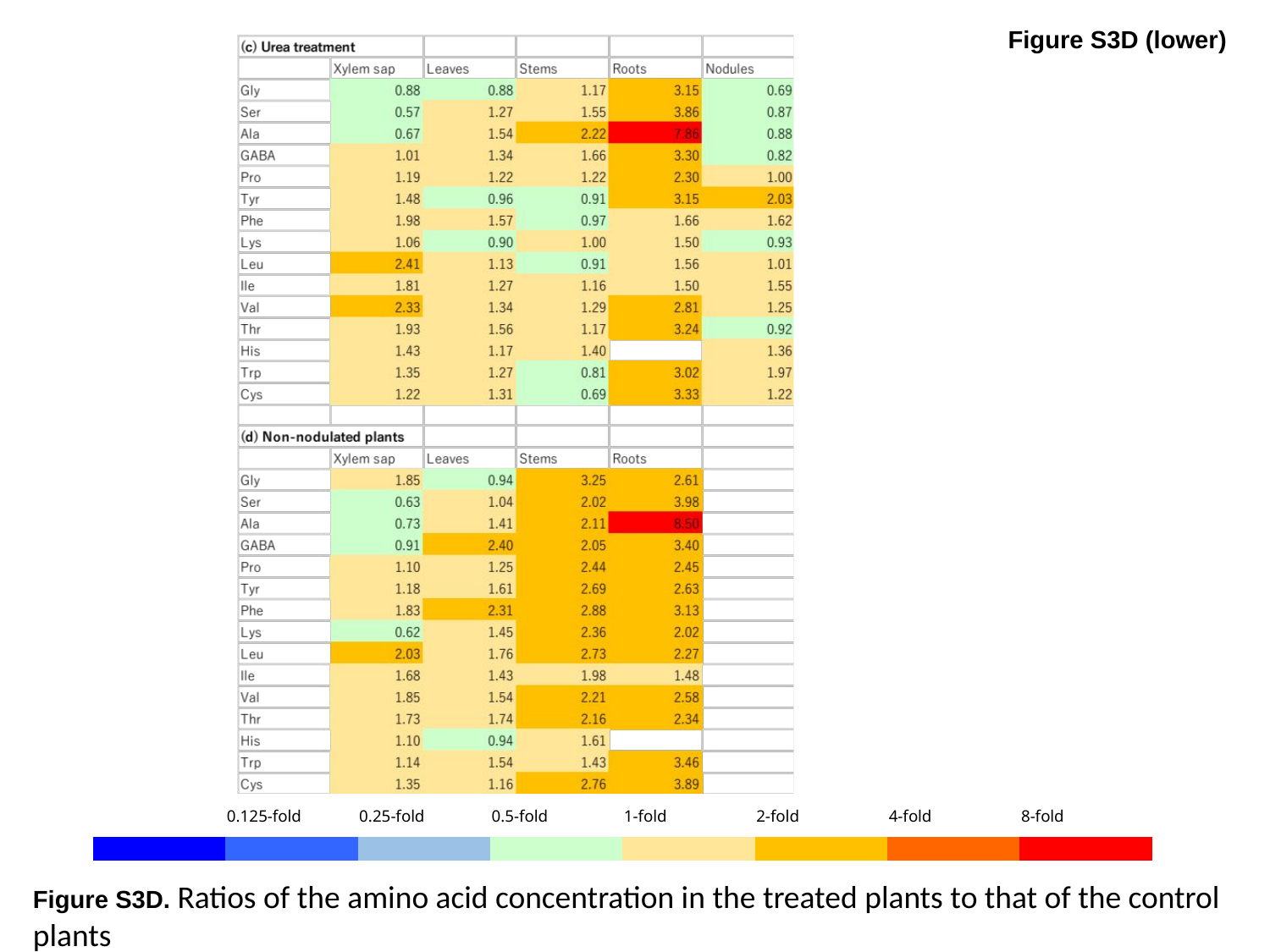

Figure S3D (lower)
| | 0.125-fold | 0.25-fold | 0.5-fold | 1-fold | 2-fold | 4-fold | 8-fold |
| --- | --- | --- | --- | --- | --- | --- | --- |
| | | | | | | | |
Figure S3D. Ratios of the amino acid concentration in the treated plants to that of the control plants

## Slide 25
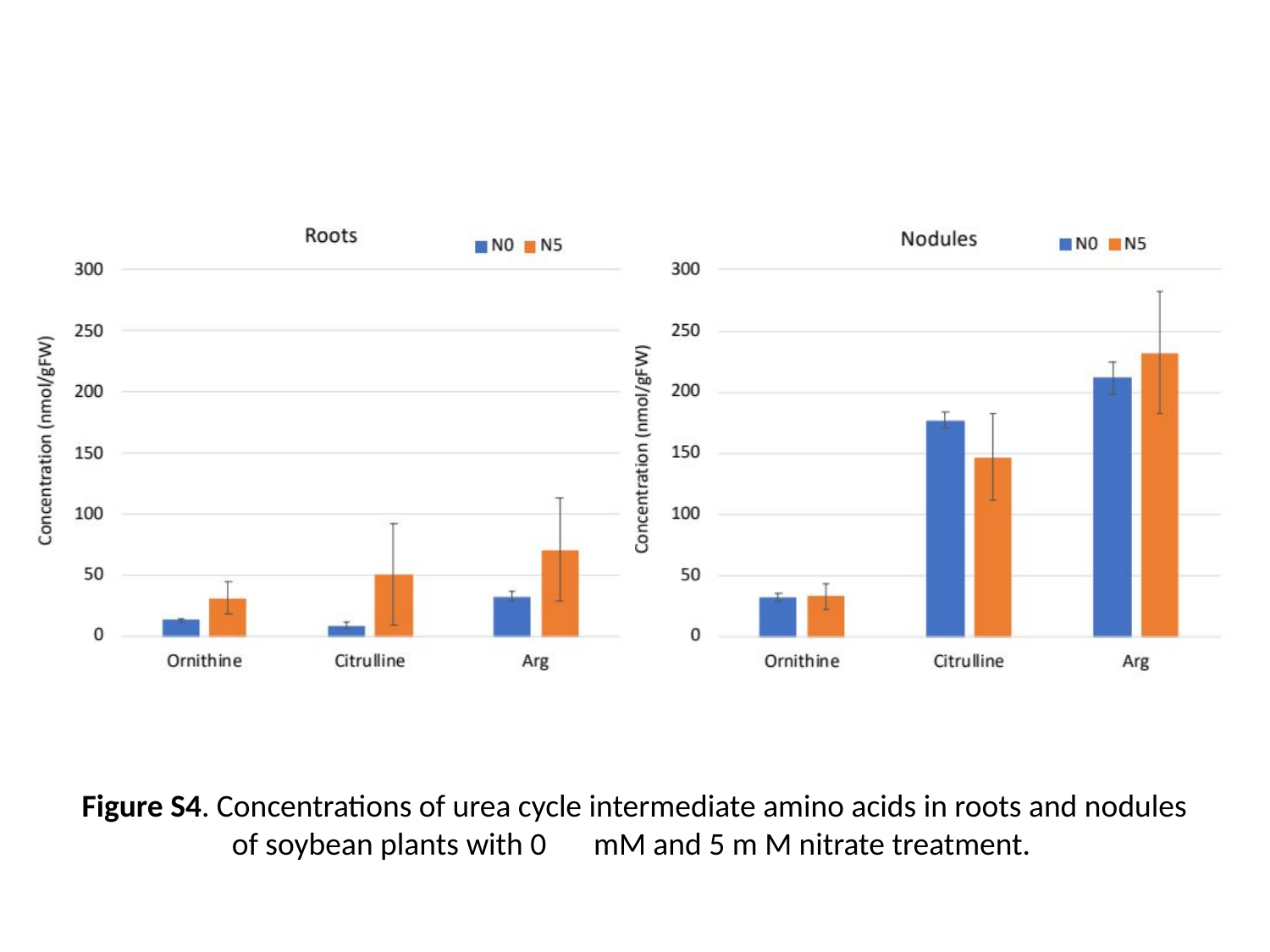

# Figure S4. Concentrations of urea cycle intermediate amino acids in roots and nodules of soybean plants with 0　mM and 5 m M nitrate treatment.

## Slide 26
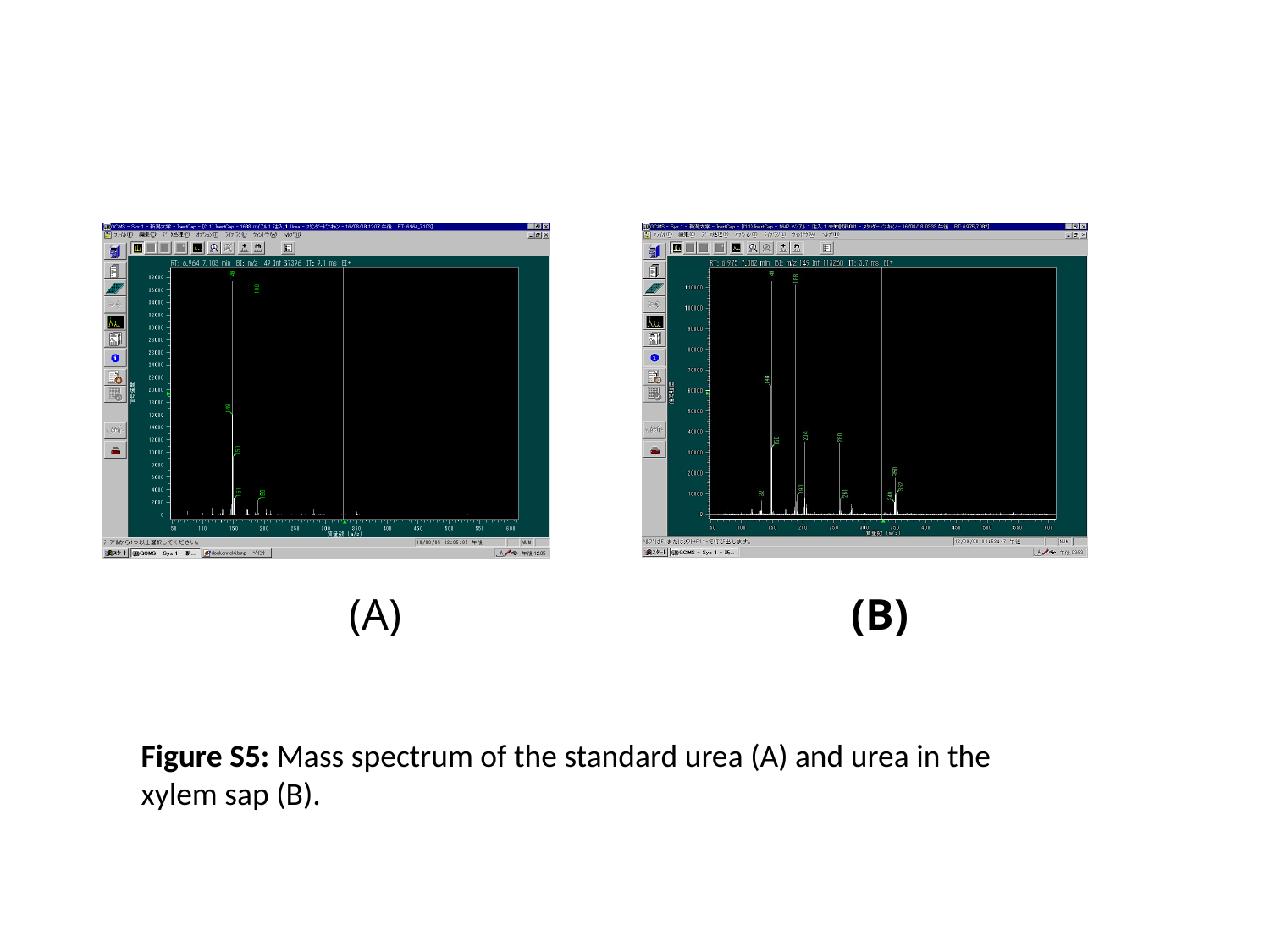

188
188
(A)
(B)
Figure S5: Mass spectrum of the standard urea (A) and urea in the xylem sap (B).
